# Alternative and Efficient Extraction Methods for Marine-Derived Compounds

**DOI:** 10.3390/md13053182

**Published:** 2015-05-21

**Authors:** Clara Grosso, Patrícia Valentão, Federico Ferreres, Paula B. Andrade

**Affiliations:** 1 REQUIMTE/LAQV, Laboratory of Pharmacognosy, Department of Chemistry, Faculty of Pharmacy, University of Porto, Rua de Jorge Viterbo Ferreira, no. 228, Porto 4050-313, Portugal; E-Mails: acfmtgrosso@ff.up.pt (C.G.); valentao@ff.up.pt (P.V.); 2 Research Group on Quality, Safety and Bioactivity of Plant Foods, Department of Food Science and Technology, CEBAS (CSIC), P.O. Box 164, 30100 Campus University Espinardo, Murcia 30100, Spain; E-Mail: federico@cebas.csic.es

**Keywords:** marine compounds, enzyme-assisted extraction, ionic liquids, microwave-assisted extraction, pressurized solvent extraction, pulsed electric field-assisted extraction, supercritical fluid extraction, ultrasound-assisted extraction, switchable solvents

## Abstract

Marine ecosystems cover more than 70% of the globe’s surface. These habitats are occupied by a great diversity of marine organisms that produce highly structural diverse metabolites as a defense mechanism. In the last decades, these metabolites have been extracted and isolated in order to test them in different bioassays and assess their potential to fight human diseases. Since traditional extraction techniques are both solvent- and time-consuming, this review emphasizes alternative extraction techniques, such as supercritical fluid extraction, pressurized solvent extraction, microwave-assisted extraction, ultrasound-assisted extraction, pulsed electric field-assisted extraction, enzyme-assisted extraction, and extraction with switchable solvents and ionic liquids, applied in the search for marine compounds. Only studies published in the 21st century are considered.

## 1. Introduction

Since the 1960’s the interest in the marine environment has greatly increased and during the last fifty years more than 20,000 compounds were isolated from marine organisms. The development of the high-resolution nuclear magnetic resonance spectrometer (NMR) in the 1970’s greatly contributed to this scenario [1] and since 1984 systematic annual reviews have been published, reporting novel marine compounds [2,3,4,5,6,7,8,9,10,11,12,13,14,15,16,17,18,19,20,21,22,23,24,25,26,27,28,29,30,31]. Before 1985, less than 100 natural products were discovered annually, but this number shifted to about 300 in 1987, remaining constant (500–600 products *per* year) in the late 1990s–2000s [1]. After 2008, the period not covered by the review by Hu *et al.* [1] that was mainly based on reviews by Faulkner [2,3,4,5,6,7,8,9,10,11,12,13,14,15,16,17,18,19] and Blunt *et al.* [20,21,22,23,24,25,26,27,28,29,30,31], Blunt and co-workers took into consideration 1011 new compounds in 2009 [28], 1003 in 2010 [29], 1152 in 2011 [30] and 1241 in 2012 [31].

Despite this huge amount of new compounds described in the literature, only a small fraction has been extensively studied, mainly for commercial/pharmaceutical purposes. This review will focus on the period after 2000 and will describe the best extraction conditions to obtain high-value compounds, such as carotenoids, fatty acids, sterols, volatile and phenolic compounds, among others. Eight alternative extraction methods, known by their high efficiency and low solvent and time consumptions, will be covered, namely microwave-assisted extraction (MAE), ultrasound-assisted extraction (UAE), supercritical fluid extraction (SFE), pressurized solvent extraction (PSE), pulsed electric field-assisted extraction (PEF), enzyme-assisted extraction (EAE), and extraction with switchable solvents and ionic liquids (ILs).

However, as we will discuss at the end of this review, the common practices are still far away from what is desired for the 21st century and, probably, more work and more environmental policies are needed to convince the research community and the industry to move one step forward and replace the conventional extractions (Soxhlet and maceration) by those described herein.

## 2. Extraction of Compounds

The preparation of natural matrices for chemical and biological analyses involves multiple steps, aiming to improve the separation of the analytes of interest to be further analyzed [32,33,34].

This review emphasizes the steps concerning the extraction of metabolites. Solid-liquid extraction (SLE) is a term used to describe the extraction of soluble constituents from a solid or semisolid matrix using suitable solvents [35]. Considering that natural matrices are complex and may contain thousands of compounds, SLE can be a long and tedious process since it is a procedure involving five sequential steps: (1) penetration of the solvent into the natural matrix; (2) solubilization of the compounds of interest; (3) transport of the solute (compounds of interest) out of the natural matrix; (4) migration of the extracted solute from the external surface of the natural matrix into the bulk solution; (5) separation of the extract and discharge of the natural matrix [36,37].

SLE is influenced by a number of important factors that must be taken into account when we need to choose the most appropriate extraction method for the natural matrix under study, namely the polarity and thermolability of the compounds of interest, the features of the solvent (toxicity, volatility, polarity, viscosity and purity), the probability of artefacts’ formation during the extraction process and the amount of biomass to be extracted [38,39].

SLE of natural products has been employed almost since the discovery of fire. In many ancient civilizations, such as the Phoenicians, Egyptians, Jews, Arabs, Indians, Chineses, Greeks, Romans, Mayans, and Aztecs, innovative extraction processes—at that time (maceration, distillation, *etc.*)—have emerged to obtain perfumes, medicines or foods [40]. Current SLE methods can be divided into two main groups: traditional and alternative ones. Traditional methods comprise Soxhlet extraction, hydrodistillation, maceration, decoction, infusion, pressing, percolation, *etc.* Although they have been used since ancient times, they are very often time-consuming and require relatively large quantities of polluting solvents, which can lead to sample contamination, losses due to volatilization during concentration steps, and environmental pollution from solvent waste [41]. For these reasons, since the late 1970’s they have been substituted by faster and more efficient/selective techniques, namely MAE, UAE, PSE, SFE, PEF, EAE, and extractions with switchable solvents and ILs [32,42].

### 2.1. Microwave-Assisted Extraction (MAE)

MAE is an efficient SLE method that takes advantage of microwave irradiation to accelerate the removal of a diversity of compounds from natural matrices [42,43,44]. In MAE, both heat and mass gradients work in the same direction, while in conventional heating they work in opposite directions [37,45]. Thus, in conventional heating, only the surface of the matrix is heated directly, and subsequent heating is by conduction from the surface to the core of the matrix particle. Conversely, MAE causes direct generation of heat within the matrix, by friction between polar molecules [45].

When a substrate containing water or other polar compounds is placed under the influence of an oscillating electric field (wavelengths: 1 mm–1 m; frequencies: 300 MHz–300 GHz; domestic and commercial systems: 2450 MHz), vibration/oscillation of polar molecules occurs, causing inter- and intra-molecular friction. This friction, together with the movement and collision of a very large number of charged ions, results in the rapid heating (in few seconds) of the matrix. Further intracellular heating leads to pressurized effects that induce cell walls and membranes to breakdown, in addition to electroporation effects. As a consequence, there is a faster transfer of the compounds from the cells into the extracting solvent [32,42,44,46].

The breakdown of cell walls is particularly important in what concerns the extraction of bioactive compounds from algae, since their cell walls are very complex [47]. Additionally, mechanical disruption techniques are also very useful to breakdown calcareous and siliceous skeletons of some hard sponges [48].

The application of microwave energy to the samples may be performed using open vessels at atmospheric pressure or closed ones, under controlled pressure and temperature. In this second case, the solvent can be heated above its normal boiling point by simply applying a defined pressure, which accelerates the mass transfer of the compounds from the natural matrix to the bulk solvent [38,41].

The higher the dielectric constant (ɛ′) of the solvent, the greater the energy absorbed by the molecules and the faster the solvent reaches the extraction temperature [32,34]. Polar solvents are better MAE extractants than non-polar ones, since ɛ′ decreases in the following order: water (ɛ′ = 78.3) >methanol (ɛ′ = 32.6) >ethanol (ɛ′ = 24.3)>acetone (ɛ′ = 20.7) >ethyl acetate (ɛ′ = 6.0) >hexane (ɛ′ = 1.9) [37].

### 2.2. Ultrasound-Assisted Extraction (UAE)

UAE significantly reduces the extraction time and increases the extraction yields of many natural matrices, due to the production of cavitation bubbles in the solvent [49]. Cavitation bubbles are produced in the liquid during the expansion phase. The negative pressure exerted by the expansion cycle exceeds the local tensile strength of the liquid [46,50]. This ability to cause cavitation depends on the characteristics of ultrasound wave, the solvent properties, and the ambient conditions [32,50,51].

After a cavitation bubble is produced, it collapses during the compression cycle, which push the liquid molecules together, and a high-speed micro-jet is created towards the matrix particle, promoting the mixture of the solvent with the matrix. The high pressure and temperature involved in this process, which can reach up to 1000 bar and up to 4726.85 °C, respectively, are responsible for an enhancement of mass transfer, since the shock wave breaks cell walls and membranes [50,51]. After cell disruption, the solvent can easily penetrate the solid particle, releasing the intracellular compounds to the bulk solvent [32,51,52,53,54].

The application of ultrasound can be divided into two distinct categories: low intensity–high frequency (100 kHz–1 MHz) and high intensity–low frequency (between 20 and 100 kHz) ultrasound, this last one being the only case that leads to the disruption of cell walls and membranes [51,55].

### 2.3. Supercritical Fluid Extraction (SFE)

The use of supercritical fluids is an alternative extraction technique that produces extracts with none or fewer polar impurities than the conventional organic liquid extracts [56]. It is considered to be a green technology, since a concentration step is most often eliminated after the extraction process [57,58].

For any solvent, its vapor/liquid equilibrium curve culminates at the critical point (CP), above which only one phase occurs. All solvents possess a CP, which is characterized by a critical temperature (*T*_c_) and a critical pressure (*P*_c_). Experimental studies using SFE are usually limited to the region of *P*_c_ < *P* ≤ 6*P*_c_ and *T*_c_ < *T* ≤ 1.4*T*_c_ [59].

The efficiency of the extraction of compounds from a natural matrix using a supercritical fluid can be much higher than that with a liquid organic solvent at atmospheric pressure. The density of the supercritical fluid (250–800 kg/m^3^) is liquid-like (800–1200 kg/m^3^) and its solvent power can be controlled by the working pressure and temperature. On the other hand, the values of diffusivity, which are high (10^−7^–10^−8^ m^2^/s), and viscosity, which are low (10^−4^–10^−3^ N·s/m^2^), are between those of gases (10^−4^–10^−5^ m^2^/s and 10^−5^–10^−4^ N·s/m^2^, respectively) and liquids (10^−8^–10^−9^ m^2^/s and 10^−3^–10^−2^ N·s/m^2^, respectively); thus, the supercritical fluid is capable of a faster and deeper penetration into the solid particles [46,57,58,59].

The most commonly used supercritical fluid is CO_2_ because it has favorable *T*_c_ and *P*_c_ (31.1 °C and 73.9 bar) that are ideal for the extraction of thermolabile compounds. In addition, supercritical CO_2_ has low viscosity, low surface tension, high diffusivity and good density and is also non-toxic, non-flammable, cheap, widely available, chemically inert under several conditions, and gaseous at normal pressure and temperature, eliminating the step of solvent evaporation after extraction [46,56,58,59,60,61]. Furthermore, CO_2_ gives a non-oxidizing atmosphere in extractions, thus preventing extracts from degradation [62].

The greatest limitation of supercritical CO_2_ is that it is not suitable to extract polar compounds. However, the addition of an organic modifier or entrainer, such as EtOH or MeOH, can greatly improve extraction efficiency [58,59].

Other solvents, such as H_2_O, MeOH, EtOH, acetone, chloroform, ethyl acetate, and toluene, are not appropriate to extract bioactive compounds, because their Tc is above 200 °C [63].

### 2.4. Pressurized Solvent Extraction (PSE)

PSE is a type of extraction that uses temperatures and pressures in the ranges of 50–200 °C and 35–200 bar, respectively, to extract desired compounds. These sets of pressure and temperature are lower than the Tc and Pc of the solvents, leading solvents to keep the liquid state. The high pressure applied causes the solvents to rise above their normal boiling point temperature and the increased temperature enhances solubility and mass transfer rate and reduces the viscosity and surface tension of the solvents. Thus, both temperature and pressure increase mass transfer rate. Water is the most widely used solvent for subcritical fluid extraction. Additionally, other solvents, such as propane and dimethyl ether (DME), can also be used for subcritical fluid extraction [46,64,65,66].

Extraction with liquefied DME is cost-efficient and environmentally friendly. Since DME is partially miscible with water, it allows the simultaneous extraction of non-polar target metabolites and the removal of water from wet matrices. Moreover, the liquefied DME (with *T*_c_ = 127 °C and *P*_c_ = 53.7 bar) is evaporated under low-pressure conditions and taken off as a gas, since its normal boiling point is low (−24.8 °C) and its saturated vapor pressure at 20 °C is 5.1 bar. Another advantage of DME over other ethers is that it is resistant to autoxidation. A common liquefied DME extraction is performed at room temperatures, at pressures around 5.1–5.9 bar. DME is afterwards evaporated from the extract simply by decompression of the collecting vessel or by heating [63,67,68,69,70].

### 2.5. Pulsed Electric Field-Assisted Extraction

Pulsed electric fields (PEFs) can be used to enhance mass transfer processes, by destroying cell membranes. A moderate PEF treatment is defined by applying a field strength of 0.5–1.0 kV/cm and treatment times in the range of 100–10,000 µs, or field strength in the range of 1–10 kV/cm and shorter times (5–100 µs) [71,72,73].

Depending on the intensity, amplitude, duration, number, and repetition frequency of the external electric pulses, reversible or irreversible pores are produced in the membranes. Irreversible pores formation is of great importance for extraction of bioactive compounds from natural matrices. Usually, treatments of electric field strength from 0.7 to 3 kV/cm, a specific energy of 1–20 kJ/kg, couple hundred of pulses, and total time duration lower than 1s are used for natural products extraction. These conditions are weaker than those required to inactivate microorganisms (15–20 kV/cm and specific energy of 40–1000 kJ/kg) [46,72,73].

The relation between the conductivity of intact and disintegrated cells is called cell disintegration index *Z*_p_. If there is no difference between the conductivity of the untreated and PEF-treated cell membrane, the *Z*_p_ value is 0. If, by contrast, *Z*_p_ = 1, this indicates the maximum of cell disintegration due to PEF treatment [72].

### 2.6. Enzyme-Assisted Extraction

Food-grade digestive enzymes, such as proteases and carbohydrases, can be used to macerate the tissues and break down the cell walls of natural matrices, releasing cell contents. This is particularly important for marine algae, since cell walls and cuticles are made up of chemically complex and heterogeneous biomolecules [64].

Temperature, pH, proportion of substrate to enzyme, type of solvent (water or buffer with appropriate pH) and agitation are important parameters to be considered [46,74]. Enzyme-assisted extraction (EAE) offers several advantages: (i) it is an ecofriendly and nontoxic process; (ii) it allows high yields of bioactive compounds to be obtained; (iii) it converts water-insoluble raw materials into water-soluble materials; and (iv) it is a relatively low-cost technology because of the use of food grade enzymes [64,75]. Table 1 displays the optimum conditions of pH and temperature of the most common enzymes used, as well as their sources.

**Table 1:** Sources, characteristics and optimum pH and temperature conditions of proteases and carbohydrases.

Enzyme	Composition and Source	Optimum pH	Optimum Temp. (°C)
Agarase [74,76]	β-Agarase from *Alteromonas beaufortensis*	6.0	55
Alcalase [74,77,78,79]	α-Endoprotease from *Bacillus licheniformis*	8.0	50
Alkaline protease [80,81]	Protease from *Bacillus lincheniformis*	8.5	55
AMG [74,77,78,79]	*exo*-1,4-α-d-Glucosidase from *Aspergillus niger*	4.5	60
Carrageenase [74,76]	κ-Carrageenase from *Cytophaga drobachiensis*	6.8	45
Celluclast [74,77,78,79]	Cellulase from *Trichoderma reesei*	4.5	50
Cellulase [74,76,81]	Cellulase from *Trichoderma viride*	3.8	50
Flavourzyme [74,77,78,79]	Endoprotease and exopeptidase from *Aspergillus oryzae*	7.0	50
Kojizyme [74,77,78,79]	Amino- and carboxylpeptidase from *Aspergillus oryzae*	6.0	40
Neutrase [74,77,78,79]	Metallo-endoprotease from *Bacillus amyloliquefaciens*	6.0	50
Neutral protease [80,81]	Protease from *Bacillus subtilis*	7.0	50
Papain [80]	Protease from *Carica papaya*	7.0	55
Protamex [74,77,78,79]	Protease from *Bacillus* sp.	6.0	40
Snailase [81]	Complex of more than 30 enzymes, including cellulase, hemicellulase, galactase, proteolytic enzyme, pectinase and β-glucuronidase, from snail	5.8	37
Termamyl [74,77,78,79]	Heat-stable α-amylase from *Bacillus licheniformis*	6.0	60
Trypsin [80,81]	Protease from porcine pancreas	8.0	37
Ultraflo [74,77,78,79]	Heat-stable multi-active β-glucanase from *Humicola insolens*	7.0	60
Umamizyme [74,79]	Endo- and exopeptidase complex from *Aspergillus oryzae*	7.0	50
Viscozyme [74,77,78,79]	Multi-enzyme complex (containing arabanase, cellulase, β-glucanase, hemicellulase and xylanase) from *Aspergillus aculeatus*	4.5	50
Xylanase [74,76]	Xylanase from *Disporotrichum dimorphosporum*	5.0	55

### 2.7. Smart Solvents: Switchable Solvents and Ionic Liquids

Switchable solvents are a smart new class of extracting solvents that are able to turn from a non-ionic form (an alcohol and an amine base) to an ionic liquid (a salt in liquid form), by bubbling CO_2_ and, by exposing to N_2_, to return to the non-ionic form. Bioactive compounds can be separated from the solvent just by adding CO_2_ [82].

Switchable-polarity solvents (SPS) display two degrees of polarity. They have low polarity while in the non-ionic form and high polarity in the ionic one, polarity ranging from that of ether/toluene to that of ethylenoglycol [82,83,84]. The low polarity form may consist of amidine/alcohol mixtures (e.g., 1,8-diazabicyclo[5.4.0]undec-7-ene (DBU)/ROH)), guanidine/alcohol mixtures (e.g., tetramethylguanidine/ROH), amidine/amine mixtures (e.g., DBU/RNH_2_), secondary amines, diamines and guanidine/acidic alcohol mixtures [84].

Switchable hydrophilicity solvents (SHS), such as several tertiary amines, are liquid solvents that are hydrophobic. However, when exposed to CO_2_, these solvents become very hydrophilic. Thus, SHS can be easily removed from the extracted natural products with carbonated water [84,85].

Ionic liquids (ILs) are designer solvents, because they can be tailor-made. They are organic liquid salts composed of large asymmetric organic cations (imidazolium, pyrrolidinium, pyridinium, ammonium or phosphonium) and several different inorganic or organic anions, such as BF4^−^, PF_6_^−^, Cl^−^, and Br^−^ anions. They are very versatile, since their polarity, hydrophobicity, viscosity and other chemical and physical properties can be selected by choosing the cationic or the anionic constituents. Moreover, they are low-melting point solvents and their non-flammable and non-volatile nature makes them an excellent choice for the development of safer processes [39,86,87]. Nonetheless, some ILs are not environmentally friendly and require a toxic and laborious purification process [88].

## 3. Most Common Marine Compounds Obtained by Alternative Extraction Processes

### 3.1. Terpenoids

Marine organisms are rich sources of bioactive terpenes with antimicrobial [89,90,91,92], anticancer [93,94,95,96,97,98] and anti-inflammatory [99] activities.

From the point of view of extraction methods, carotenoids are the most studied class of compounds. Their usefulness is extensive, having a variety of applications in the food industry as colorants in foodstuffs, for instance, astaxanthin, zeaxanthin, and lutein [100,101,102], being also used for animal feeding in aquaculture [95,101].

Regarding human health, some carotenoids (α- and β-carotene and β-cryptoxanthin) display provitamin A activity [95,103]. They also act as potent antioxidants, anti-inflammatory and cardiovascular protectors, such as astaxanthin and fucoxanthin [95,104], although at high oxygen pressures they behave as pro-oxidants [105]. Lutein and zeaxanthin act as preventive antioxidants against age-related macular degeneration (AMD) and age-related cataracts [100,102]. Moreover, the nervous system is rich in both unsaturated fats and iron (with strong pro-oxidative properties), which make tissues particularly prone to oxidative damage. Thus, because of their antioxidant potential, carotenoids could fight neurodegenerative diseases [104]. Epidemiological studies have also suggested that the intake of β-carotene, astaxanthin, cantaxanthin, and zeaxanthin is inversely correlated with cancer prevalence [95,103,104].

#### 3.1.1. Monoterpenes and Sesquiterpenes

Gao *et al.* [106] performed SFE to extract halogenated monoterpenes from red seaweed *Plocamium cartilagineum* (Linnaeus) P.S. Dixon. Different temperatures (40–100 °C), pressures (250–400 bar) and % of entrainer (2%–10% of methanol) were tested and several compounds were extracted: (3*R*,4*S*)3-methyl-3,4,8-trichloro-1,5(*E*),7(*E*)-octatriene-7-al; (3*R*,4*S*)7-bromochloromethyl-3-methyl-3,4,8-trichloro-1,5(*E*),7(*E*)-octatriene; (3*R*,4*R*)7-dichloromethyl-3-methyl-3,4,8-trichloro-1,5(*E*),7(*Z*)-octatriene; (3*R*,4*S*)7-dichloromethyl-3-methyl-3,4,8-trichloro-1,5(*E*),7(*Z*)-octatriene; (3*R*,4*R*)1-bromo-7-dichloromethyl-3-methyl-3,4,8-trichloro-1(*E*),5(*E*),7(*Z*)-octatriene; (3*R*,4*S*)1-bromo-7-dichloromethyl-3-methyl-3,4,8-trichloro-1(*E*),5(*E*),7(*Z*)-octatriene; (3*R*,4*S*,7*S*)3,7-dimethyl-1,8,8-tribromo-3,4,7-trichloro-1(*E*),5(*E*)-octadiene; and (3*R*,4*S*,7*S*)1,8-dibromo-3,7-dimethyl-3,4,7-trichloro-1(*E*),5(*E*)-octadiene. Concerning the influence of pressure and temperature, above 300 bar/40 °C the concentration of (3*R*,4*S*)3-methyl-3,4,8-trichloro-1,5(*E*),7(*E*)-octatriene-7-al in the SFE extracts was higher than that at 250 bar/40 °C. By contrast, the concentration of (3*R*,4*S*)7-bromochloromethyl-3-methyl-3,4,8-trichloro-1,5(*E*),7(*E*)-octatriene at 250 bar/40 °C was higher than that above 300 bar/40 °C. Moreover, pure CO_2_ yielded more halogenated monoterpenes than CO_2_ mixed with ethanol, since the mixture also extracted other kinds of compounds. It was found that the recoveries of (3*R*,4*S*)3-methyl-3,4,8-trichloro-1,5(*E*),7(*E*)-octatriene-7-al and of (3*R*,4*S*)7-bromochloromethyl-3-methyl-3,4,8-trichloro-1,5(*E*),7(*E*)-octatriene using SFE reached 238% and 218%, respectively, of the same monoterpenes obtained from conventional solvent extraction with hexane. However, recoveries of compounds (3*R*,4*R*)7-dichloromethyl-3-methyl-3,4,8-trichloro-1,5(*E*),7(*Z*)-octatriene, (3*R*,4*S*)7-dichloromethyl-3-methyl-3,4,8-trichloro-1,5(*E*),7(*Z*)-octatriene, (3*R*,4*R*)1-bromo-7-dichloromethyl-3-methyl-3,4,8-trichloro-1(*E*),5(*E*),7(*Z*)-octatriene, (3*R*,4*S*)1-bromo-7-dichloromethyl-3-methyl-3,4,8-trichloro-1(*E*),5(*E*),7(*Z*)-octatriene and (3*R*,4*S*,7*S*)3,7-dimethyl-1,8,8-tribromo-3,4,7-trichloro-1(*E*),5(*E*)-octadiene in relation to the conventional extraction were 133%, 100%, 78%, 37%, and 47%, respectively.

The volatiles of *Gloiopeltis tenax* (Turner) Decaisne, a marine red algae, were obtained by SFE at 300 bar, 45 °C and CO_2_ + ethanol [107]. Twenty three compounds belonging to different chemical classes were identified: terpenes ((−)-thujopsene, cedrol, (+)-cuparene, α-curcumene, (−)-β-bisabolene and α-zingiberene), fatty acids, sterols, among others. This extract revealed antioxidant activity and antimicrobial potential against *Staphyloccocus aureus*, *Enterococcus faecalis*, *Pseudomonas aeruginosa* and *E. coli* [107].

Johnson *et al.* [108] performed a PSE of the sponge *Cacospongia mycofijiensis* Kakou, Crews and Bakus (samples 07327A and 07327G) with three different solvents (hexane, dichloromethane and methanol), at 117 bar, 110 °C, as well as traditional solvent extraction with dichloromethane. Since three unrecognizable metabolites were detected in the PSE dichloromethane fractions as well as in the traditional extract, they further exhaustively extracted the compounds from 07327A with the traditional technique, affording the sesquiterpenes, aignopsanoic acid A, methyl aignopsanoate A, and isoaignopsanoic acid A, together with non-terpenoid compounds: latrunculol A (a latrunculin that has been pursued as a preclinical antitumor candidate [109]), fijianolide B (a polyketide macrolide with taxol-like microtubule-stabilizing activity [110]) and latrunculin A (an inhibitor of actin polymerization [111]). Aignopsanoic acid A and methyl aignopsanoate A were active against the parasite *Trypanosoma brucei* [108]. Later, the same authors [109] analyzed the PSE dichloromethane fraction obtained from sample 07327F, identifying and isolating 16 compounds, from which 12 were already known, including latrunculins, fijianolides, mycothiazole, the aignopsanes, and sacrotride A. From the four new compounds identified, two were derivatives of the aignopsane class, aignopsanoic acid B and aignopsane ketal*.*

Several mono and sesquiterpenes were extracted by MAE-hydrodistillation from the brown algae *Dictyopteris membranaceae*, namely α-cubebene, α-copaene, β-bourbonene, β-cubebene, germacrene D, aromadendrene, azulene, sativene, epi-bicyclosesquiphellandrene, δ-cadinene, α-calacorene, axenol, 1,10-di-*epi*-cubebol, α-amorphene, and vulgarol B [112].

#### 3.1.2. Diterpenes

Phytol was isolated from the brown algae *Dictyopteris membranaceae* by SFE at 91 bar/40 °C, 1.8 g/min of CO_2_ flow rate and 35 min of extraction time (5 min static time + 30 min dynamic time) [112].

The meroditerpenes (+)-isojaspic acid, cacospongin D and jaspaquinol were isolated from the marine sponge *Cacospongia* sp. They were recovered from fractions P3, P4 and P5 obtained with PSE performed with hexane, dichloromethane, and methanol. Their antimicrobial activity was evaluated against *Bacillus subtilis*, *Escherichia coli*, and *Staphylococcus epidermidis*, being active against this last one [113].

#### 3.1.3. Carotenoids and Other Pigments

Table 2, Table 3, Table 4 and Table 5 list the best extraction conditions for marine carotenoids using MAE [43,114], UAE [53,60,61,65,114,115,116,117], SFE [53,56,57,60,61,62,69,70,100,116,118,119,120,121,122,123,124,125,126,127,128] and PLE [65,69,70,115,129,130,131,132]. When several extraction conditions were tested, the best ones are marked with a symbol (§). In the majority of the cases, carotenoids are co-extracted with other pigments, such as chlorophylls. In Table 4, we report the conditions of pressure and temperature that allow obtaining the highest ratio of carotenoids/chlorophylls (Car/Chl) in order to have SFE extract richer in carotenoids.

In comparison with conventional methods, alternative extraction methodologies claim to reduce extraction time, energy consumption as well as solvent amounts used in the extraction. For instance, compared to conventional extraction methods, the use of microwaves and UAE accelerated the extraction of pigments from the diatom *Cylindrotheca closterium* (Ehrenberg) Reimann and J.C.Lewin, since extraction yields obtained after 60 min by soaking (either at room temperature or high temperature) were achieved in a few minutes using MAE and UAE [114]. However, sometimes the recovery of the compounds of interest is lower than that obtained with maceration and Soxhlet extraction. For *Nannochloropsis* sp., the total pigments yield obtained with SFE (300 bar, 40 °C, 140 min) corresponded to 70% recovery compared to cold extraction (performed until the supernatant was colorless) with ethyl acetate and acetone [120]. Compared with the conventional extraction with ethanol (room temperature, 120 min), SFE (400 bar, 40 °C, 180 min) from the seaweed *Undaria pinnatifida* (Harvey) Suringar allowed also the recovery of ≈80% of total fucoxanthin. However, a pretreatment by microwave before SFE shifted the recovery to almost 120% [56].

**Table 2:** Microwave-assisted extraction of carotenoids and chlorophylls.

Species	Compounds	Biomass (g)	Solvent	Microwave Power (W)	*F* (MHz)	*T* (°C)	Time (min)	B:S Ratio (g/mL) ^a^	Yield of Pigments at Best Extraction Conditions
**Microalgae**									
***Cylindrotheca closterium* (Ehrenberg) Reimann & J.C.Lewin** [114]	Fucoxanthin and chlorophyll *a*	0.050 (dried)	Acetone	25–100 **50 §**		22 (VMAE ^b^), 56 (MAE) **MAE §**	3–15 **5 §**	1:600	Fucoxanthin (4.2 μg/mg dried biomass) Chlorophyll *a* (8.7 μg/mg dried biomass)
***Dunaliella tertiolecta* Butcher** [114]	β,β-Carotene, chlorophyll *a* and chlorophyll *b*	0.050 (dried)	Acetone	25–100 **50 §**		22 (VMAE ^b^), 56 (MAE) **MAE §**	3–15 **5 §**	1:600	β,β-Carotene (~1.2 μg/mg dried biomass) Chlorophyll *a* (~4.5 μg/mg dried biomass) Chlorophyll *b* (~1.4 μg/mg dried biomass)
**Macroalgae**									
***Laminaria japonica* Areschoug** [43]	Fucoxanthin	fresh	EtOH	300	2450	60	10	1:15	Fucoxanthin (0.05 μg/mg dried biomass)
***Sargassum fusiforme* (Harvey) Setchell** [43]	Fucoxanthin	2 (dried)	EtOH	300	2450	60	10	1:15	Fucoxanthin (0.02 μg/mg dried biomass)
***Undaria pinnatifida* (Harvey) Suringar** [43]	Fucoxanthin	2.0 (dried)	MeOH, EtOH, acetone, DMSO and hexane:EtOH, 1:1 **EtOH §**	300–500 **300 §**	2450	30–60 **60§**	5–15 **10§**	1:5–1:15 **1:15§**	Fucoxanthin (1.1 μg/mg dried biomass)

^a^ B:S ratio—Biomass: solvent ratio; ^b^ VMAE—vacuum microwave-assisted extraction (performed at 0.267 bar).

**Table 3:** Ultrasound-assisted extraction of carotenoids and chlorophylls.

Species	Compounds	Biomass (g)	Solvent	Ultrasound Power (W)	*F* (kHz)	*T* (°C)	Time (min)	B:S Ratio (g/mL) ^a^	Yield of Pigments at Best Extraction Conditions
**Microalgae**									
***Cylindrotheca closterium* (Ehrenberg) Reimann & J. C. Lewin** [114]	Fucoxanthin and chlorophyll *a*	0.050 (dried)	Acetone	4.3–12.2 **12.2§**		8.5	3–15 **5 §**	1:600	Fucoxanthin (4.5 μg/mg dried biomass) Chlorophyll *a* (5.0 μg/mg dried biomass)
***Dunaliella salina* (Dunal) Teodoresco** [53,61]	Carotenoids and chlorophylls	0.1 (dried)	MeOH, DMF ^b^ **DMF §**				3	1:50	Carotenoids (27.7 μg/mg dried biomass) Chlorophylls (3.1 μg/mg dried biomass) Car/Chl = 8.9
***Dunaliella tertiolecta* Butcher** [114]	β,β-Carotene, chlorophyll *a* and chlorophyll *b*	0.050 (dried)	Acetone	4.3–12.2 **12.2 §**		8.5	3–15 **5 §**	1:600	β,β-Carotene (~1.2 μg/mg dried biomass) Chlorophyll *a* (~4.8 μg/mg dried biomass) Chlorophyll *b* (~1.3 μg/mg dried biomass)
***Nannochloropsis gaditana* L. M. Lubián** [60]	Carotenoids and chlorophyll *a*	0.2 (dried)	MeOH				10	1:25	Carotenoids (0.8 μg/mg dried biomass) Chlorophyll *a* (18.5 μg/mg dried biomass) Car/Chl = 0.04
***Nannochloropsis gaditana* L. M. Lubián** [61]	Carotenoids and chlorophyll *a*	0.2 (dried)	MeOH, DMF **DMF §**				10	1:25	Carotenoids (6.9 μg/mg dried biomass) Chlorophyll *a* (41.5 μg/mg dried biomass) Car/Chl = 0.2
***Phaeodactylum tricornutum* Bohlin** [115]	Fucoxanthin	0.5 (dried)	EtOH ^c^		70	23	30	1:50	Fucoxanthin (16.0 μg/mg dried biomass)
***Synechococcus* sp.** [116]	Carotenoids and Chlorophyll *a*	0.1 (dried)	MeOH				10	1:50	Carotenoids (1.4 μg/mg dried biomass) Chlorophyll *a* (4.1 μg/mg dried biomass) Car/Chl = 0.3
***Synechococcus* sp.** [61]	Carotenoids and chlorophyll *a*	0.1 (dried)	MeOH, DMF **DMF §**				10	1:50	Carotenoids (3.3 μg/mg dried biomass) Chlorophyll *a* (9.6 μg/mg dried biomass) Car/Chl = 0.3
**Macroalgae**									
***Laminaria japonica* Areschoug** [65]	Carotenoids and chlorophyll *a*	0.5 (dried)	MeOH				30	1:20	Carotenoids (0.3 μg/mg dried biomass) Chlorophyll *a* (3.0 μg/mg dried biomass) Car/Chl = 0.1
**Crustaceans**									
***Penaeus brasiliensis* Latreille + *Penaeus paulensis* Pérez Farfante** [117]	Astaxanthin and β-criptoxanthin	5.0 (dried)	EtOH ^d^		55		10	1:30	Total carotenoids content (0.04 μg/mg extract)

^a^ B:S ratio—Biomass: solvent ratio; **^b^** DMF—*N*,*N′*-dimethylformamide; ^c^ Selection of the solvent using normal maceration (acetone, ethanol, water, *n*-hexane, ethyl acetate); ^d^ Pretreatment—cooking/drying/milling.

**Table 4:** Supercritical fluid extraction of carotenoids and chlorophylls.

Species	Compounds	Biomass (g)	Co-Solvent	*P* (bar)	*T* (ºC)	Time (min)	CO_2_ Flow Rate (g/min)	Yield of Pigments at Best Extraction Conditions
**Bacteria**								
***Paracoccus zeaxanthinifaciens*** [100]	Zeaxanthin	1.0 (dried)	Acetone, water, EtOH, MeOH, isopropyl alcohol **MeOH §**	100–500 **300 §**	40–80 **40 §**	40–160 + 20 # **160 + 20 §**	0.36–1.79 ** 0.90 §**	Zeaxanthin (65% of recovery)
**Microalgae**								
***Chlorococcum littorale* M. Chihara, T. Nakayama & I. Inouye** [118]	Violaxanthin, neoxanthin, antheraxanthin, lutein, zeaxanthin, β-carotene, chlorophylls	0.7 (dried)	EtOH 10% ^a^	300	60	180	0.65	Xantophylls (~0.6 μg/mg dried biomass) β-carotene (~0.2 μg/mg dried biomass)
***Dunaliella salina* (Dunal) Teodoresco** [62]	α-Carotene and β-carotene (13-*cis*, all-*trans*, 15-*cis*, 9-*cis*)	1.0 (dried)	No	182.7–437.3 **437.3 §**	9.8–45.2 * **27.5 §**	10 + 90 #		α-Carotene (9.1 μg/mg extract) 13-*cis*-β-Carotene (4.2 μg/mg extract) All-*trans*-β-Carotene (12.4 μg/mg extract) 15-*cis*-β-Carotene (5.9 μg/mg extract) 9-*cis*-β-Carotene (15.0 μg/mg extract)
***Dunaliella salina* (Dunal) Teodoresco** [53]	Carotenoids and chlorophylls	0.1 (dried)	No	100–500 **300—Car § 500—Chl §**	40–60 **60 §**	15 + 180#	0.20	Carotenoids (14.9 μg/mg dried biomass) Chlorophylls (0.4 μg/mg dried biomass) Car/Chl = 156.3 ^b^
***Dunaliella salina* (Dunal) Teodoresco** [61,119]	Carotenoids and chlorophylls	0.1 (dried)	EtOH 5%	200–500 **400 §**	40–60 **60 §**	15 + 180#	0.20	Carotenoids (9.6 μg/mg dried biomass) Chlorophylls (0.7 μg/mg dried biomass) Car/Chl = 84.5 ^b^
***Nannochloropsis* sp.** [120]	Violaxanthin/neoxanthin, astaxanthin, vaucheriaxanthin, lutein/zeaxanthin, canthaxanthin, β-carotene and chlorophyll *a*	1.25 (dried)	EtOH 5%–20% or CO_2_ + EtOH 20% **CO_2_ + EtOH 20% §**	125–300 **300 §**	40, 60 **40 §**	≈120–360 **~140 §**	0.35–0.62 **0.62 §**	Total pigments (1 μg/mg dried biomass)
***Nannochloropsis gaditana* L. M. Lubián** [60]	Carotenoids and chlorophyll *a*	0.2 (dried)	No	100–500 **400 §**	40–60 **60 §**	15 + 180#	0.20	Carotenoids (0.3 μg/mg dried biomass) Chlorophyll *a* (2.2 μg/mg dried biomass) Car/Chl = 1.4 ^c^
***Nannochloropsis gaditana* L. M. Lubián** [61,119]	Carotenoids and chlorophyll *a*	0.2	EtOH 5%	200–500 **500 §**	40–60 **60 §**	15 + 180 #	0.20	Carotenoids (2.9 μg/mg dried biomass) Chlorophyll *a* (0.4 μg/mg dried biomass) Car/Chl = 500 ^d^
***Nannochloropsis oculata* (Droop) D. J. Hibberd** [121]	Zeaxanthin, fucoxanthin, neoxanthin, β-cryptoxanthin, lutein, α-carotene and β-carotene	10 (dried)	EtOH, dichloromethane, toluene or soybean oil **EtOH §**	250–350 **350 §**	50		0.04	Zeaxanthin (1.1 μg/mg dried biomass)
***Synechococcus* sp.** [122]	Zeaxanthin, β-cryptoxanthin, echinenone, β-carotene and chlorophyll *a*	0.1 (dried)	No	200–500 **500—total Car and zeaxanthin § 358—β-carotene § 454—β-cryptoxanthin §**	40–60 **60—total Car and zeaxanthin § 50—β-carotene § 59—β-cryptoxanthin §**	240	0.20	Total carotenoids (~2.8 μg/mg dried biomass) β-Carotene (~1.5 μg/mg dried biomass) β-Cryptoxanthin (~0.1 μg/mg dried biomass) Zeaxanthin (~0.6 μg/mg dried biomass)
***Synechococcus* sp.** [116]	Carotenoids and chlorophyll *a*	0.1 (dried)	No	100–500 **300—Car § 500—Chl §**	40–60 **50—Car § 60—Chl §**	15 + 180 #	0.20	Carotenoids (1.5 μg/mg dried biomass) Chlorophyll *a* (0.7 μg/mg dried biomass) Car/Chl = 101.3^c^
***Synechococcus* sp.** [61,119]	Carotenoids and chlorophyll *a*	0.1 (dried)	EtOH 5%	200–500 **300 §**	40–60 **50—Car § 60—Chl §**	15 + 180 #	0.20	Carotenoids (1.9 μg/mg dried biomass) Chlorophyll *a* (2.2 μg/mg dried biomass) Car/Chl = 147.5^e^
**Macroalgae**								
**Undaria pinnatifida (Harvey) Suringar** [56]	Fucoxanthin	5 (dried)	No	200–400 **400 §**	25–60 * **40 §**	180	0.16–2.32 **2.32 §**	Fucoxanthin (~ 1.2 μg/mg dried biomass)
***Undaria pinnatifida* (Harvey) Suringar** [123]	Fucoxanthin	10 (dried)	EtOH	80–300 **200 §**	30–60 **50 §**	60	28.17	Fucoxanthin (7.5×10^−6^ μg/mg dried biomass)
***Undaria pinnatifida* (Harvey) Suringar** [69,70]	Fucoxanthin	3 (dried)	EtOH 1.7%–17% **1.7%, 3.2% §**	100–400 **400 §**	40–70 **60 §**	~240	0.005	Fucoxanthin (1.0 μg/mg dried biomass)
**Crustaceans**								
***Callinectes sapidus* Rathbun** [124]	Astaxanthin	10–25 (dried) **25 §**	EtOH 10%	295–345 **340 §**	45–65 **45 §**		3.4–4.8 ^f^	Astaxanthin (0.012 μg/mg dried biomass)
***Euphausia superba* Dana** [57]	Astaxanthin	35 (dried)	No	150–250 **250 §**	35–45 **45 §**	150	22	Astaxanthin (0.09 μg/mg extract)
***Farfantepenaeus paulensis* Latreille** [126]	Astaxanthin	7 (dried)	No	200–400 **370 §**	40–60 **43 §**	20 + 200 #	2.5	Astaxanthin (0.02 μg/mg dried biomass)
***Farfantepenaeus paulensis* Latreille** [125]	Astaxanthin	7 (dried)	EtOH 10%	300	50	20 + 200 #	2.50–5.00 **5.00 §**	Astaxanthin (0.03 μg/mg dried biomass)
***Litopenaeus vannamei* Boone** [127]	Astaxanthin and β-carotene	(dried)	EtOH 20%	150–300, increment 50 bar/30 min	40, 50 **50 §**	120	20	Astaxanthin (0.04 μg/mg dried biomass) β-Carotene (0.03 μg/mg dried biomass)
***Penaeus monodon* Fabricius** [128]	Astaxanthin, lutein and β-carotene	5 (dried)	water, MeOH, EtOH, MeOH:water (1:1), EtOH:water (1:1), EtOH:water (0.7:0.3), MeOH:water (0.7:0.3) **EtOH §**	200	60	15 + 120#		Astaxantin (0.01 μg/mg dried biomass) Lutein (0.0005 μg/mg dried biomass) β-Carotene (0.002 μg/mg dried biomass)

* Some extractions were performed in the subcritical region; # (static time + dynamic time); ^a^ Best Car/Chl without co-solvent, flow rate 0.65 g/min; Best Car/Chl: at 500 bar, 40 °C ^b^; at 200 bar, 60 °C ^c^; at 300 bar, 40 °C ^d^; at 200 bar, 50 °C ^e^; ^f^ CO_2_ + ethanol mixture flow rate in L/min.

**Table 5:** Pressurized liquid extraction of carotenoids and chlorophylls.

Species	Compounds	Biomass (g)	Solvent	*P* (bar)	*T* (°C)	Time (min)	Flow Rate (g/min)	Yield of Pigments at Best Extraction Conditions
**Microalgae**								
***Dunaliella salina* (Dunal) Teodoresco** [129]	α-Carotene, 13-*cis*-β-carotene, all-*trans*-β-carotene, 15-*cis*-β-carotene, 9-cis-β-carotene	2.0 (dried)	Hexane, EtOH, water **EtOH §**	103.4	40–160 **160 §**	5–30 **30 §**	static	α-Carotene (1.6 μg/mg extract) 13- *cis*-β-Carotene (2.0 μg/mg extract) all-*trans*-β-Carotene (6.6 μg/mg extract) 15-*cis*-β-Carotene (4.5 μg/mg extract) 9-*cis*-β-Carotene (9.5 μg/mg extract)
***Phaeodactylum tricornutum* Bohlin** [115]	Fucoxanthin	0.5 (dried)	EtOH	103.4	100	30	static	Fucoxanthin (16.5 μg/mg dried biomass)
**Macroalgae**								
***Eisenia bicyclis* (Kjellman) Setchell** [130]	Fucoxanthin	2, 4 (fresh) **2§**	EtOH 50%, 100% **EtOH 90% §**	68.95–172.37 **103.4 §**	40, 100 **110 §**	5, 15 **5 §**	static	Fucoxanthin (0.4 μg/mg)
***Laminaria japonica* Areschoug** [65]	Fucoxanthin, lutein, zeaxanthin, β-carotene and chlorophyll *a*	0.5 (dried)	EtOH + co-solvent (R134a ^a^, 2%–6%) **EtOH + R134a, 4.73% §**	50–170 **170§**	30–60 **51§**	15 + 50 #	10	Carotenoids (0.2 μg/mg dried biomass) Chlorophylls (2.3 μg/mg dried biomass) Car/Chl =0.1
***Undaria pinnatifida* (Harvey) Suringar** [132]	Fucoxanthin	620, 800 (wet)	DME with or without 10% EtOH **DME + EtOH 10% §**	40	60			Fucoxanthin (0.06 μg/mg wet biomass) ^b^
***Undaria pinnatifida* (Harvey) Suringar** [69,70]	Fucoxanthin	4.4 (wet)	DME	5.9	25	<43	≈0.3	Fucoxanthin (0.4 μg/mg dried biomass)
**Crustaceans**								
***Euphausia pacifica* Hansen** [131]	Astaxanthin	1.0^c^ (dried)	R134a ^a^	30–150 **100 §**	30–70 **60 §**	10–50 **30 §**	2–10 **6 §**	Astaxanthin (0.2 μg/mg dried biomass)

^#^ (static time + dynamic time); ^a^ R134a—1,1,1,2-tetrafluoroethane; ^b^ With enzyme (alginase lyase) pre-treatment; ^c^ Optimum moisture content of 5.5% (tested from 5.5%–63.6%).

Similar results were obtained in other studies, in which SFE was compared with conventional extraction methods. Sánchez-Camargo *et al.* [125] observed a recovery of astaxanthin from *Farfantepenaeus paulensis* Latreille equal to 57.9% using SFE (300 bar, 50 °C, 220 min, CO_2_ + ethanol 10%), which was similar to that of acetone extraction performed until no further pigment was extracted by the solvent (63.3%), but inferior than that obtained by maceration with 60% (v/v) *n*-hexane:isopropyl alcohol. Using the same matrix, Sánchez-Camargo *et al.* [126] recovered 39% of astaxanthin by SFE (370 bar, 43 °C, 220 min), which was lower than the recovery attained by maceration with 60% (v/v) *n*-hexane:isopropyl alcohol. Better results were obtained for pigments extraction from the crustacean *Euphausia superba* Dana [57], SFE (250 bar, 45 °C, 150 min) yielding 86% of astaxanthin recovery in comparison with Soxhlet extraction performed during 12 h. Almost 100% of carotenoids recovery was obtained from other crustacean, *Penaeus monodon* Fabricius, applying 200 bar, 60 °C, 135 min and ethanol as co-solvent, showing that SFE is as effective as maceration carried out with a mixture of acetone and methanol (7:3, v/v) at room temperature [128]. Concerning PSE of astaxanthin from the crustacean *Euphausia pacifica* Hansen using 1,1,1,2-tetrafluoroethane, with this method it was recovered 87.14% of astaxanthin in less time (30 min) than with Soxhlet using dichloromethane (180 min) [131]. Mezzomo *et al.* [117] compared maceration, Soxhlet, UAE and hot and cold oil extraction for astaxanthin recovery from the crustaceans *Penaeus brasiliensis* Latreille + *Penaeus paulensis* Farfante. Although UAE did not achieve the highest extraction yield, it was considered a good alternative, since it greatly reduced the extraction time.

In addition, the comparison of different alternative methods also gave the conclusion that they have different efficiencies, as well as selectivities. For carotenoids and chlorophylls extraction, most of the results showed that the best extraction yields were obtained when UAE was carried out, but SFE was a more efficient method for the recovery of carotenoids, since higher Car/Chl ratios were obtained. These results suggest that the SFE is more selective than UAE [53,60,61,116].

Billakanti *et al.* [132] compared subcritical DME + ethanol 10% extraction of fucoxanthin from *Undaria pinnatifida* performed with and without an enzymatic pre-treatment (alginate lyase enzyme, pH 6.2, 37 °C). Alginate is a gelling polysaccharide found in great abundance as part of the cell wall and intracellular material in brown seaweeds and alginate lyase catalyzes the degradation of alginate by a β-elimination mechanism, targeting the glycosidic 1→4 *O*-linkage between monomers [133]. Enzyme pre-treatment increased the extraction yield by 50% of fucoxanthin from *Undaria pinnatifida* (from 0.03 to 0.06 μg/ g wet biomass), which corresponds to 75% of the total recovery obtained using conventional ethanol extraction plus enzymatic pre-treatment or extraction with chloroform:methanol:water (1:1:1) [132].

Kanda *et al.* [69] tested different alternative methods (liquefied DME, SFE with CO_2_ and SFE with CO_2_ + ethanol) and Soxhlet extraction with ethanol to recover fucoxanthin from *Undaria pinnatifida*. Besides the disadvantage of the time consumed during the traditional extraction method (12 h), the yield of fucoxanthin was also lower (0.05 μg/mg of biomass) than those obtained with pressurized techniques, namely SFE-CO_2_ at 400 bar and 60–70 °C (180 min, 0.06 μg of fucoxanthin/mg of biomass), liquefied DME (43 min, 0.4 μg of fucoxanthin/mg of biomass) and SFE-CO_2_ + ethanol at 400 bar, 60 °C (180 min, 1.0 μg of fucoxanthin /mg of biomass).

The use of ILs recently demonstrated advantages to extract astaxanthin from shrimp waste [134]. 1-Butyl-3-methylimidazolium bromide ([C_4_MIM][Br]), 1-butyl-3-methylimidazolium chloride ([C_4_MIM][Cl]), 1-butyl-3-methylimidazolium methylsulfate ([C_4_MIM][MS]), 1-butyl-3-methylimidazolium tetrafluoroborate ([C_4_MIM][BF_4_]), 1-ethyl-3-methylimidazolium tetrafluoroborate ([C_2_MIM][BF_4_]), 1-hexyl-3-methylimidazolium tetrafluoroborate ([C_6_MIM][BF_4_]) and 1-propylamine-3-methylimidazolium bromide ([C_3_NH_2_MIM][Br]) were selected for the extractions (solid/liquid ratio, g/mL =1:10; ultrasonic power = 75 W; time = 60 min; room temperature). Simultaneously, UAE, in the same operational conditions, but with different conventional solvents (methanol, ethanol, hexane, ethyl acetate, acetone, dichloromethane, and water), was also performed, ethanol being the most efficient extractant (≈0.045 μg/mg dried shrimp waste). The highest astaxanthin yield was obtained with ILs-UAE for the extraction performed with [C_3_NH_2_MIM][Br], *i.e.*, ≈0.080 μg/mg dried shrimp waste.

### 3.2. Free and Bound Fatty Acids

Fatty acids are ubiquitous in biological systems and a wide range of bioactivities are attributed to them. The intake of both monounsaturated fatty acids (MUFA) and ω3 polyunsaturated fatty acids (PUFA) has been associated with reduced cardiovascular diseases risk [135,136,137,138,139]. On the other hand, excessive amounts of ω6 PUFA and a very high ω6:ω3 ratio promote the pathogenesis of many diseases, such as cardiovascular, cancer, inflammatory and autoimmune diseases [136,140].

Several studies have also shown the beneficial effects of MUFA and PUFA in the brain. Regarding PUFA, γ-linolenic acid possesses neuroprotective and anti-inflammatory properties [59,141]. Docosahexaenoic acid and arachidonic acid are considered to be essential for proper visual and neurological development of infants [136,142]. However, the consumption of large amounts of ω-3 fatty acids by pregnant women or nursing infants could have harmful consequences [143].

Rheumatoid arthritis is a chronic inflammatory autoimmune disease of the joints and bones. Since marine eicosapentaenoic acid and docosahexaenoic are substrates for LOX and COX, like arachidonic acid, these fatty acids are known to inhibit arachidonic acid metabolism [144]. The lipid-rich SFE extracts from the mussels *Perna canaliculus* Gmelin powder (Lyprinol) and *Mytilus coruscus* Gould, showed significant *in vivo* anti-inflammatory effect on rat models for chronic arthritis [145,146].

The ability of fatty acids to interfere with bacterial growth and survival has also been known for several decades [147,148,149,150,151].

A recent review about extraction of lipids from terrestrial, freshwater and marine microalgae for biodiesel production, using expeller, MAE, UAE, conventional solvent extraction, SFE and ILs, has been published [152]. Studies concerning SFA, MUFA, and PUFA extracted from marine organisms using MAE [153,154,155], UAE [54,153,156], SFE [80,120,121,126,137,140,142,147,157,158,159,160,161,162,163], PSE [164,165,166,167] and EAE [80,163,168,169] are gathered in Table 6, Table 7, Table 8 and Table 9. When several extraction conditions were tested, the best ones were marked with a symbol (§).

**Table 6:** Microwave-assisted and ultrasound-assisted extraction of fatty acids from microalgae.

Species	Compounds	Biomass (g)	Solvent	Microwave/Ultrasound Power (W)	*F* (MHz)	*T* (°C)	Time (min)	B:S Ratio (g/mL) ^a^	Yield of Oil at Best Extraction Conditions
**Microwave-assisted extraction**									
***Dunaliella tertiolecta* Butcher** [153]	C16:0, C18:0, C18:1, C18:2, C18:3	0.2 (dried)	Chloroform/MeOH (2:1)	280–560 **490 §**			2–3.33 **2.66 §**	1:100–1:150 **1:100 §**	Total oil extracted (57.0%)
***Nannochloropsis* sp.** [154]	C12:0, C13:0, C14:0, C14:1, C15:0, C16:0, C16:1, C17:0, C17:1, C18:0, C18:1, C18:2, C18:3 (2 isomers), C20:0, C20:1, C20:2, C20:3, C20:4, C20:5, C22:0, C22:1, C22:2, C22:6, C23:0, C24:0,C24:1	1 (dried = 3.3 wet)	Chloroform/EtOH (1:2), methyl soyate 20%, 40% in EtOH **Methyl soyate 40% in EtOH §**	1200	2450	80–120 **120 §**	15		Total fatty acids (45.24%, dried weight) Total saturated fatty acids (~30%, dried weight) Total unsaturated fatty acids (~15%, dried weight)
***Nannochloropsis* sp.** [155]	C14:0, C16:0, C18:0, C18:1ω9t, C18:1ω9c, C18:2ω6t, C18:2ω6c	15 ^b^	Isopropanol + hexane, MeOH + chloroform, MeOH + dichloromethane, MeOH:chloroform:water (25:12.5:5) + chloroform with 1.5% sodium sulfate **MeOH + chloroform §**	500		65	5 + 5	*	Total oil extracted (9%, v/w)
***Tetraselmis* sp.** [155]	C14:0, C16:0, C18:0, C18:1ω9t, C18:1ω9c, C18:2ω6t, C18:2ω6c	15 ^b^	Isopropanol + hexane, MeOH + chloroform, MeOH + dichloromethane, MeOH:chloroform:water (25:12.5:5) + Chloroform with 1.5% sodium sulfate **Isopropanol + hexane §**	500		65	5 + 5	*	Total oil extracted (8%, v/w)
**Ultrasound-assisted extraction**									
***Dunaliella tertiolecta* Butcher** [153]	C16:0, C18:0, C18:1, C18:2, C18:3	0.2 (dried)	Chloroform/MeOH (2:1)	320–400 **370 §**			4–6 **5 §**	1:100–1:150 **1:125 §**	Total oil extracted (45.94%)
***Nannochloropsis oculata* (Droop) D. J. Hibberd** [156]	C14:0, C16:0, C16:1ω7, C16:1ω9, C16:2ω6, C16:3ω3, C18:0, C18:1ω7, C18:1ω9, C18:2ω6, C18:3ω6, C20:4ω6, C20:5ω3	100 (fresh) (% dry weight content 5–30) **% dry weight content 5 §**	Solvent-free	300–1000 **1000 §**		35	5–30 **30 §**		Total oil extracted (0.21%)
***Tetraselmis suecica* Kylin(Butch)** [54]	C12:0, C14:0, C16:0, C16:1, C16:4,C18:0, C18:1, C18:2, C18:3	700 mL of wet culture (2 g/L)	Water	500–1000 **1000 §**					Total fatty acid content (70 μg/mg dried biomass)

^a^ B:S ratio—biomass:solvent ratio; ^b^ 15 mL concentrated wet marine microalgae; * Hara and Radin method: 15 mL of wet biomass in 20 mL isopropanol + 30 mL hexane; Folch *et al.* method: 15 mL of wet biomass in 25 mL MeOH + 50 mL chloroform; Chen *et al.* method: 15 mL wet biomass in 25 mL MeOH + 50 mL dichloromethane; Bligh and Dyer method: 15 mL of wet biomass in MeOH:chloroform:water (12.5: 12.5: 5) + chloroform: 1.5% sodium sulfate (12.5:12.5).

**Table 7:** Supercritical fluid extraction of fatty acids.

Species	Compounds	Biomass (g)	Co-Solvent	*P* (Bar)	*T* (°C)	Time (min)	CO_2_ Flow Rate (g/min)	Yield of Oil at Best Extraction Conditions
**Microalgae**								
***Chaetoceros muelleri* Lemmermann** [147]	C12:0, C14:0, C16:0, C16:1, C16:2, C16:3, C18:0, C18:1, C18:2, C18:3, C20:5ω3, C22:5ω3	1 (dried) + 0.2% EtOH	EtOH	200–400 **400 §**	40–80 **40 §**	15 + 45 #		Total oil extracted (3.9%)
***Chlorococcum* sp.** [157]	C14:0, C16:0, C16:1, C16:1t, C16:2, C17:0, C18:0, C18:1, C18:2, C20:5	20 (dried) 8 (wet paste) **8 §**		Two setps: first (100–300); second (300–500)	60, 80 **60 §**	120	0.7	Total oil extracted (71 μg/mg wet biomass)
***Crypthecodinium cohnii* (Seligo) Javornicky** [142]	12:0, 14:0, 16:0, 16:1, 18:0, 18:1ω9, 22:5ω3, 22:6ω3	30 (dried)	No	200–300 **300 §**	40, 50 **50 §**	180	10	Total oil extracted (86 μg/mg dried biomass)
***Nannochloropsis* sp.** [158]	C14:0, C16:0, C18:0, C14:1, C16:1ω7, C18:1ω9, C18:2ω6, C18:3ω3, C20:4ω6, C20:5ω3,C22:5ω3, C22:6ω3	180 (dried)	No	400–700 **550 and 700 §**	40, 55 **55 §**	360	166.7	Total lipids (≈250 μg/mg dried biomass)
***Nannochloropsis sp.* [120]**	C13:0, C14:0, C15:0, C16:0, C16:1, C17:1, C18:0, C18:1, C18:2, C18:3, C20:4, C20:5	1.25 (dried)	EtOH 5%–20% or CO2 + 20% EtOH **CO2 + EtOH 20% §**	125–300 **300 §**	40, 60 **40 §**	≈120–360 **~140 §**	0.35–0.62 **0.62 §**	Total lipids (450 μg/mg dried biomass)
***Nannochloropsis oculata* (Droop) D. J. Hibberd** [121]	C16:0, C16:1, C18:1, C18:2, C20:4, C20:5	10 (dried)	Ethanol or dichloromethane **No co-solvent §**	350	50		0.02–0.04 **0.04 §**	Total lipids (441.2 μg/mg of extract)
***Nannochloropsis oculata* (Droop) D. J. Hibberd** [159]	C14:0 (2 isomers), C14:1ω5, C15:0 (3 isomers), C15:1ω8, C16:0 (2 isomers), C16:1ω7, C16:1ω5, C16:2ω6, C16:2ω4, C16:3ω6, C16:3ω3, C17:0 (3 isomers), C17:1, C18:0 (2 isomers), C18:1ω9, C18:1ω7, C18:1ω5, C18:2ω6, C18:2ω4, C18:3ω6, C18:3ω3, C18:4ω3, C19:0, C20:0, C20:1ω9, C20:1ω7 , C20:2ω6, C20:3ω6, C20:4ω6, C20:4ω3, C20:5ω3, C22:0, C22:6ω3	2–13 (dried)	No	400	60	120	6.7–8.3	Total oil extracted (300 μg/mg dried biomass)
***Nannochloropsis oculata* (Droop) D. J. Hibberd** [160]	C14:0, C14:1ω5, C15:0 (3 isomers), C15:1ω8, C16:0 (2 isomers), C16:1ω7, C16:1ω5, C16:2ω6, C16:2ω4, C16:3ω6, C16:3ω3, C17:0 (3 isomers), C17:1, C18:0 (2 isomers), C18:1ω9, C18:1ω7, C18:1ω5, C18:2ω6, C18:2ω4, C18:3ω6, C18:3ω3, C18:4ω3, C19:0, C20:0, C20:1ω9, C20:1ω7 , C20:2ω6, C20:3ω6, C20:4ω6, C20:4ω3, C20:5ω3, C22:0, C22:6ω3	10 (dried)		400	60	60–120 ^a ^**120 §**	8.3	Total oil extracted mainly composed by triglycerides
***Nannochloropsis granulata* B. Karlson & D. Potter** [161]	C12:0, C14:0, C16:0, C16:1ω7, C18:0, C18:1ω7, C18:1ω9(c&t), C18:2ω6c, C20:4ω6, C20:5ω3	350 (dried)	No	350–550 **350 §**	50–90 **70 §**	180–360 **270 §**	100	Total lipids (28.5 μg/mg dried biomass) Total fatty acids (18.23 μg/mg dried biomass)	
***Nannochloropsis salina* D. J. Hibberd** [140]	C14:0, C16:0, C16:1ω9, C16:1ω7c, C16:2ω6, C16:3ω3, C16:4ω3, C18:0, C18:1ω9, C18:2ω6, C18:3ω3, C18:4ω3, C18:5ω3, C20:5ω3	0.5 (dried)	EtOH 5%	300	45	90	6.7	Total oil extracted (304.0 μg/mg dried biomass) Total MUFA (33.9% of total lipids) Total PUFA (5.7% of total lipids)
***Schizochytrium limacinum* Honda et Yokochi** [162]	C14:0, C15:0, C16:0, C18:0, C18:1, C18:2ω6, C20:5ω3, C22:6ω3	5 (dried) ^b^	Ethanol	150–400 **350 §**	30–60 **40 §**	30–180		Total lipids (33.9%) Total C22:6ω3 (27.5%)
**Mollusca**								
***Dosidicus gigas* Orbigny** [137]	C14:0, C16:0, C16:1, C18:0, C18:1ω9, C18:1ω7, C18:2ω6, C18:3ω6, C18:3ω3, C18:4ω3, C20:1ω9, C20:3ω6, C20:4ω6, C20:5ω3, C22:1ω11, C22:1ω9, C22:4ω6, C22:5ω3, C22:6ω3, C24:1	100 (dried)	No	250	40	180	166.7	Total oil extracted (~150 μg/mg dried biomass) Total fatty acids (691.0 μg/mg oil) Total ω3 fatty acids (284.0 μg/mg oil) Total ω6 fatty acids (29.0 μg/mg oil)
***Patinopecten yessoensis* Jay** [163]	C13:0, C14:0, C16:0, C16:1ω7, C16:2ω6, C16:3ω3, C16:4ω3, C18:0, C18:1ω9, *trans*-C-18:1ω9, C18:2ω5, C18:2ω6, C18:4ω3, C20:1ω7, C20:1ω9, C20:5ω5, C22:6ω3	30 (dried)	No	280	50	80	0.75	Total oil extracted (185.6 μg/mg dried biomass) Total SFA (22.4% of total extract) Total MUFA (19.6% of total extract) Total PUFA (56.7% of total extract) EPA+DHA (39.4% of total extract)
**Crustaceans**								
***Farfantepenaeus paulensis* Latreille** [126]	C10:0, C12:0, C14:0, C15:0, C16:0, C16:1ω7, C17:0, C17:1, C18:0, C18:1ω9t, C18:1ω9, C18:2ω6, C18:2ω6t, C18:3 (two isomers), C18:4ω3, C20:0, C20:1ω11, C20:4ω6, C20:5ω3, C22:1ω9, C22:5ω3, C22:6ω3, C24:0	7 (dried)	No	200–400 **370 §**	40–60 **57 §**	20 + 200 #	2.5	Total oil extracted (2.0%) Total PUFA (29.8% of total extract) Total ω3 fatty acids (18.3% of total extract)
**Echinoderms**								
***Strongylocentrotus nudus* A. Agassiz** [80]	C14:0, C14:1ω3, C15:0, C16:0, C16:1ω5, C16:1ω7, C16:1ω9, C18:0, C18:1ω7, *trans*-C18:1ω9, C18:1ω11, C18:2ω6, C18:2ω7, C18:3ω6, C19:1ω9, C20:0, C20:1ω7, C20:1ω9, C20:2ω6, C20:2ω7,10, C20:3ω3, C20:4ω3, C20:4ω6, C20:5ω3, C22:1ω9, C22:6ω3	30 (dried)	No	280	50	80	0.59	Total oil extracted (53.7%) Total SFA (28.1% of total extract) Total MUFA (29.1% of total extract) Total PUFA (33.3% of total extract)
**Fishes**								
***Hoplostethus atlanticus* Collett** [137]	C14:0, C16:0, C16:1, C18:0, C18:1ω9, C18:1ω7, C18:2ω6, C18:3ω6, C20:1ω9, C20:3ω6, C20:5ω3, C22:1ω11, C22:1ω9, C22:6ω3, C24:1	100 (dried)	No	250	40	180	166.7	Total oil extracted (~650 μg/mg dried biomass) Total fatty acids (388.0 μg/mg fish oil) Total ω3 fatty acids (8.0 μg/mg fish oil) Total ω6 fatty acids (11.0 μg/mg fish oil)
**Fishes**								
***Merluccius capensis* Castelnau–*Merluccius paradoxus* Franca** [137]	C14:0, C16:0, C16:1, C18:0, C18:1ω9, C18:1ω7, C18:2ω6, C18:3ω6, C18:3ω3, C18:4ω3, C20:1ω9, C20:3ω6, C20:4ω6, C20:5ω3, C22:1ω11, C22:1ω9, C22:4ω6, C22:5ω3, C22:6ω3, C24:1	100 (dried)	No	250	40	180	166.7	Total oil extracted (~150 μg/mg dried biomass) Total fatty acids (595.0 μg/mg fish oil) Total ω3 fatty acids (132.0 μg/mg fish oil) Total ω6 fatty acids (19.0 μg/mg fish oil)
***Salmo salar* L.** [137]	C14:0, C16:0, C16:1, C18:0, C18:1ω9, C18:1ω7, C18:2ω6, C18:3ω6, C18:3ω3, C18:4ω3, C20:1ω9, C20:3ω6, C20:4ω6, C20:5ω3, C22:5ω3, C22:6ω3, C24:1	100 (dried)	No	250	40	180	166.7	Total oil extracted (~400 μg/mg dried biomass) Total fatty acids (789.0 μg/mg fish oil) Total ω3 fatty acids (100.0 μg/mg fish oil) Total ω6 fatty acids (108.0 μg/mg fish oil)

^#^ (static time + dynamic time); ^a^ Two types of drying: drying under air flow (120 min of extraction) and freeze-drying (60 min of extraction); ^b^ With and without pretreatment with UAE with ethanol.

**Table 8:** Pressurized liquid extraction of fatty acids from microalgae.

Species	Compounds	Biomass (g)	Solvent	*P* (bar)	*T* (°C)	Time (min.)	Flow Rate (g/min)	Yield of Oil at Best Extraction Conditions
***Nannochloropsis oculata* (Droop) D. J. Hibberd** [164]	C8:0, C10:0, C12:0, 14:0, C14:1, C15:0, C16:0, C16:1, C16:2, C16:3, C16:4, C17:0, C17:1, C18:0, C18:1, C18:2ω6, C18:3ω3, C18:3 ω6, C20:3ω6 C20:4ω6, C20:5ω3, C22:0, C22:1	~3–6	Hexane, hexane/2-PrOH (2:1 v/v) and EtOH (96 v/v) with BHT (0.05 g/L) **Hexane §**	~100–120	60	48	static	Total PUFA (57.0 μg/mg biomass) Total C20:5ω3 (37.0 μg/mg biomass)
***Nannochloropsis salina* D. J. Hibberd** [165]	C12:0, C14:0, C16:0, C16:1, C18:0, C18:1ω9c, C18:2ω6c, C18:3ω6, C18:3ω3, C20:3ω6, C20:4ω6, C20:5ω3	3.30–11.70 (CH) ^a^ 10–30 (MWH) ^a^ **CH: 7.5 § MWH: 25 §**	water	24.5 (CH) 21.5 (MWH)	180–273 (CH) 168–220 (MWH) **CH: 220 § MWH: 205 §**	9.89–35.11 (CH) 10–30 (MWH) **CH: 25 § MWH: 25 §**	static	Total oil extracted (~490 μg/mg dried biomass for CH and 300–400 μg/mg dried biomass dried weight for MWH)
***Phormidium* sp.** [166]	C16:0, C16:1ω7, C16:2, C18:0	2 (dried)	*n*-Hexane, *n*-hexane:EtOH (1:1), limonene, limonene:EtOH (1:1) **Limonene:EtOH (1:1) §**	207	50–200 **200 §**	15	static	Total oil extracted (68.0 μg/mg dried biomass)
***Phormidium* sp.** [167]	C16:0, C16:1ω7 C18:2ω6	1 (dried)	Hexane, EtOH, water **EtOH §**	103.4	50–200 **200 §**	20	static	Total oil extracted (409.0 μg/mg dried biomass)

^a^ biomass loading (%wt. of biomass/wt. of water); CH—conventional heating; MWH—microwave-assisted heating.

**Table 9:** Enzyme-assisted extraction of fatty acids.

Species	Compounds	Biomass (g)	Solvent	Enzyme	*T* (°C)	Time (min)	Yield of Oil at Best Extraction Conditions
**Mollusca**							
***Patinopecten yessoensis* Jay** [163]	C13:0, C14:0, C16:0, C16:1ω7, C16:2ω6, C18:1ω9, C18:2ω5, C18:2ω6, C18:4ω3, C20:1ω7, C20:1ω9, C20:5ω5, C22:6ω3	50 (dried)	Water (pH = 7); lipids recovered with hexane	papain	50	240	Total SFA (23.7% of total extract) Total MUFA (19.5% of total extract) Total PUFA (55.4% of total extract)
**Echinoderms**							
***Strongylocentrotus nudus* A. Agassiz** [80]	C14:0, C14:1ω3, C15:0, C16:0, C16:1ω5, C16:1ω7, C16:1ω9, C18:0, C18:1ω7, *t*-C18:1ω9, C18:1ω11, C18:2ω6, C18:2ω7, C18:3ω6, C19:1ω9, C20:0, C20:1ω7, C20:1ω9, C20:2ω6, C20:2ω7,10, C20:3ω3, C20:3ω6, C20:4ω3, C20:4ω6, C20:5ω3, C22:1ω9, C22:6ω3	10	Water (pH =7, 8 or 8.5); lipids recovered with hexane	Papain, neutral protease, alkaline protease, trypsin	40, 50 or 55	180	Total SFA (25.2%–26.4% of total extract) ^b^ Total MUFA (28.8%–30.4% of total extract) ^b^ Total PUFA (34.7%–37.4% of total extract) ^b^
**Fishes**							
***Salmo salar* L.** [168]	C14:0, C14:1ω9, C15:0, C16:0, C16:1ω7, C16:1ω9, C16:3ω3, C16:4ω3, C17:0, C18:0, C18:1ω7, C18:1ω9, C18:1ω11, C18:2ω6, C18:3ω3, C18:4ω3, C20:0, C20:1ω7, C20:1ω9, C20:1ω11, C20:2ω6, C20:3ω6, C20:4ω3, C20:4ω6, C20:5ω3, C22:1ω9, C22:1ω11, C22:6ω3	200,000	- (pH = 6.5)	Protamex	55 and then 90	60	Total SFA (24.6%–26.7% of total fraction) ^c^ Total MUFA (31.1%–36.8% of total fraction) ^c^ Total PUFA (35.0%–39.8% of total fraction) ^c^
**Salmon heads** [169]	C14:0, C16:0, C18:0, C16:1ω7, 18:1ω9, C20:1ω9, C18:2ω6, C20:4ω6, C18:3ω3, C18:4ω3, C20:4ω3, C20:5ω3, C22:5ω3, C22:6ω3	10000	- (pH = 7 or 7.5);	Neutrase, alcalase, flavourzyme; **Alcalase §** PUFA concentration with lipase	45, 50 or 55	120	Total SFA (19.9%–20.2% of total fatty acids) ^d^ Total MUFA (33.3%–33.5% of total fatty acids) ^d^ Total PUFA (46.5% of total fatty acids) ^d^

^a^ Combination of EAE with UAE; ^b^ Range of percentages corresponds to different enzymes used for EAE; ^c^ Range of percentages corresponds to different fractions: insoluble fraction, emulsion fraction, aqueous fraction and salmon oil; ^d^ Range of percentages corresponds to permeate and permeate re-esterified.

Converti *et al.* [170] compared different procedures, namely classical extraction with petroleum ether, a Soxhlet extraction with the same solvent, the Folch method (mixture of chloroform and methanol), UAE combined with Folch method and UAE with petroleum ether, to extract lipids from *Nannochloropsis oculata*. The highest lipid yield was achieved with UAE combined with Folch method (243 μg lipids/mg dry biomass). The overall lipid fraction mainly consisted of palmitic acid (60%).

Qv *et al.* [153] compared the extraction efficiency of MAE (2.66 min) and UAE (5 min) to extract fatty acids from the green algae *Dunaliella tertiolecta* Butcher and concluded that MAE achieved higher yields. By comparing MAE (methyl soyate 40% in ethanol, 120 °C, 15 min) with Soxhlet extraction (8 h), Iqbal *et al.* [154] obtained 115% recovery of a lipid extract from *Nannochloropsis* sp. with the alternative methodology. Nobre *et al.* [120] extracted lipids from *Nannochloropsis* sp. using SFE (300 bar, 40 °C, 140 min, CO_2_ + ethanol 20%) and obtained similar yields as for the Soxhlet extraction (6 h) [120]. Teo and Idris [155] compared conventional heating with microwave heating to extract lipids from the microalgae *Tetraselmis* sp. and observed that microwave was more efficient, allowing the heating temperature to be reduced from 100 to 65 °C.

Sometimes, alternative methods can be less efficient but more selective and less-time consuming than the conventional ones. SFE (300 bar, 50 °C) was less efficient than the conventional method for lipids extraction from the microalgae *Crypthecodinium cohnii* (Seligo) Javornicky, but the Bligh and Dyer method extracted a range of lipid compounds with different polarities, while CO_2_ was more selective towards the non-polar ones. The higher DHA percentage of total fatty acids was achieved by SFE [142].

Another example of selectivity was shown by Sánchez-Camargo *et al.* [126]. The SFE extracts contained approximately 40% of saturated fatty acids, against 32% in the extract obtained by Soxhlet extraction with petroleum ether. An increase of the pressure resulted in increased PUFA content, with a maximum of 30% at 370 bar and 57 °C, tending to the composition of the oil obtained by the Soxhlet extraction. The effect of temperature at high pressures (370 bar) on the PUFA concentration was also important, being higher at 57 °C than at 43 °C. The saturated/unsaturated fatty acid ratio and the PUFA/MUFA ratio decreases and increases, respectively, with the increase of pressure [126]. Therefore, by combining different sets of pressure and temperature, it is possible to obtain extracts with different compositions.

Moreover, Zhou *et al.* [163] compared the efficiency of EAE and SFE for lipid extraction from the scallop *Patinopecten yessoensis* Jay and concluded that SFE extracted higher amounts than EAE (78.3% and 60.6%, respectively). Both extractions were less efficient than Soxhlet performed with ethyl ether during 10 h, although much less time-consuming (EAE—240 min; SFE—80 min). Similar results were found for the sea urchin *Strongylocentrotus nudus* A. Agassiz, reinforcing the idea that Soxhlet extraction is more efficient but much more time and solvent consuming than EAE (performed with papain, or neutral protease, or alkaline protease or trypsin) and SFE [80].

Lipids were also successfully extracted from wet samples of *Nannochloropsis gaditana* L. M. Lubián and *Tetraselmis suecica* (Kylin) Butcher with SPS. The SPS chosen was N,N-dimethylcyclohexylamine (DMCHA), a lipophilic tertiary amine. Compared to the traditional hot extraction with chloroform and methanol, higher yields were obtained, probably due to additional non-volatile compounds extracted with DMCHA [171]. However, as the authors pointed out, a major concern regarding the use of amines as extracting solvents is the possible toxic effects to humans and environmentally valuable organisms. Indeed DMCHA possesses significant water solubility (18 g L^−1^) and adverse biological effects (e.g., 50% of the maximum adverse effect, EC_50_, is 88.5 mg L^−1^ towards *Desmodesmus* sp.) [171].

After EAE of compounds from *Salmo salar* L. with Protamex, different fractions were obtained by two-phase and three-phase separation procedures, namely insoluble fraction, salmon oil, emulsion fraction and aqueous fraction. These fractions were characterized in terms of amino acids, lipids and fatty acids, and ash content. Regarding lipids and fatty acids, the salmon oil consisted almost of triacylglycerols and was the only fraction that did not contain phospholipids. The fatty acid profile of all fractions was similar, but salmon oil was slightly poorer in docosahexaenoic acid (a ω3 fatty acid) than the other fractions [168].

Hao *et al.* [172] compared the efficiency of SFE (316 bar, 10 min) and EAE (with neutral protease, 40 °C, 120 min) in the extraction of sturgeon oil. SFE oil was superior in terms of oil yield and quality, since it contained lower acid value (AV) and peroxide value (PV) and the color was bright yellow. The fatty acid composition of the extracts obtained with both methodologies was similar, being mainly composed of oleic (35.03%–36.96%), palmitic (16.36%–19.39%) and linoleic (16.20%–17.62%) acids. However, it was observed that SFE may involve the co-extraction of isomers of benzene, which are considered to be environmental contaminants.

### 3.3. Sterols

Seaweeds contain appreciable amounts of sterols, which form an important group among the steroids and may display numerous biological activities, such as anti-diabetic, anti-cancer, anti-inflammatory and antioxidant effects. However, an accurate determination of the biological activity of individual sterols is currently difficult because pure compounds are expensive and barely available. Fucosterol and 24-methylenecholesterol are sterols characteristic of brown algae that afford several health benefits to humans [173].

Fucosterol, isolated from the brown alga *Pelvetia siliquosa* C. K. Tseng and C. F. Chang, was orally administered to streptozotocin-induced diabetic rats and proved to have anti-diabetic properties, since it caused a decrease in serum glucose concentrations and exhibited capacity to inhibit sorbitol accumulation in the lenses. Similarly, when orally administered to epinephrine-induced diabetic rats, fucosterol also caused an inhibition of blood glucose level and glycogen degradation [174,175]. The antioxidant activity of this sterol was also observed *in vivo* [176].

Xiao *et al.* [173] tested the best MAE conditions to obtain fucosterol and 24-methylenecholesterol from *Undaria pinnatifida* (Harvey) Suringar. Two grams of dried sample were mixed with ethanolic KOH (0.5–2.0 mol/L) and different conditions of microwave power (300–500 W), solid:liquid ratio (1:10–1:30 g/mL), irradiation time (10–30 min) and extraction temperature (30–70 °C) were evaluated. According to the authors, the optimum MAE conditions were as follow: 1.5 mol/L of ethanolic KOH, 500 W microwave power, 1:20 g/mL of solid:liquid ratio, 70 °C of extraction temperature, and 20 min of irradiation time. Under these conditions, the extraction yields of fucosterol and 24-methylenecholesterol were 1.21 μg/mg (dried biomass) and 0.16 μg/mg (dried biomass), respectively.

Three sterols were isolated from the red algae *Gloiopeltis tenax* (Turner) Decaisne, by SFE at 300 bar, 45 °C and CO_2_ + ethanol, namely, cholesta-4,6-dien-3β-ol, cholesterol and cholesta-3,5-dien-7-one [107].

Becerra *et al.* [177] tested the antileishmanial activity of fucosterol recovered from the seaweed *Lessonia vadosa* Searles by SFE (180 bar, 50 °C) and PSE (hexane and EtOAc, 40 or 60 °C). The results obtained showed that fucosterol may represent a new lead scaffold for antileishmanial drugs.

Fifteen sterols from scallop (*Patinopecten yessoensis* Jay) were extracted by Soxhlet with ethyl ether. Most of them were also present in the papain extract and in the extract obtained by SFE, with the exception of desmosterol, cholestanol, D:A-Friedooleanan-3-ol,(3β)- and cholest-5-en-3-ol,24-propylidene-,(3β)- [163]. Cholesterol, desmosterol, ergosta-5,24-dien-3-ol(3β) and fucosterol were found in different extracts obtained from the sea urchin *Strongylocentrotus nudus*, either by Soxhlet extraction (10h) or by SFE (280 bar, 50 °C, 80 min) or EAE (180 min) with papain, or neutral protease, or alkaline protease or trypsin [80].

### 3.4. Miscellaneous

Ralifo *et al.* [178] isolated pyrroloacridine alkaloids (plakinidine A, plakinidine E) from the sponge *Plakortis quasiamphiaster* Díaz and van Soest, together with amphiasterin B1 and amphiasterin B2, using PSE with dichloromethane. The cytotoxicity against human and murine cancer cell lines, as well as the antimicrobial activity against the yeast *Saccharomyces cerevisiae* were evaluated, revealing that plakinidine A was potent and selective against H-116 cells, while no activity was observed for the other three compounds. On the other hand, only plakinidine E displayed antimicrobial activity.

Johnson *et al.* [179] compared PSE with a conventional extraction to obtain bioactive compounds from sponges. Five marine sponges (*Cacospongia mycofijiensis* Kakou, Crews and Bakus, *Auletta constricta* Pulitzer-Finali, *Zyzzya fuliginosa* Carter, *Fascaplysinopsis reticulata* Hentschel and *Jaspis coriacea* Carter) were selected and twelve major metabolites were reported from these abovementioned sponges: fijianolide B, latrunculin A, mycothiazole, milnamide C, jasplakinolide, makaluvamines C, H, D and J, fascaplysin and bengamides A and B. PSE was performed at 22–27 or 100 °C, 117 bar, 3 cycles of 5 min static time, first with water and then with a sequence of less polar solvents (hexane, dichloromethane, and methanol). On the other hand, conventional extraction was performed with three successive extractions using 500 mL methanol (24 h), followed by a sequence of water and dichloromethane extractions. Water extract was further partitioned with *sec*-butanol. The dichloromethane fraction was re-extracted three times with hexane and partitioned with 90% aqueous methanol. The aqueous methanol layer was further partitioned with dichloromethane. The authors concluded that PSE was much more efficient than the conventional method in terms of time consumption and extraction yield.

Anaëlle *et al.* [180] compared the total phenolic content of different extracts obtained from the seaweed *Sargassum muticum* (Yendo) Fensholt using conventional, PSE (120 °C, 103 bar, 20 min), SFE (152 bar, 60 °C, CO_2_ + ethanol and 90 min) and centrifugal partition extraction. Different combination of solvents were used for both conventional and alternative extraction methods, ethyl acetate:water (50:50) being the most efficient mixture to extract phenolic compounds, while CO_2_:ethanol (88:12) was the least effective one. Concerning the antioxidant activity of all extracts, ethyl acetate:water (50:50) extract was the most active one, PSE extract being less active than the correspondent conventional one. SFE extract was inactive.

Heo *et al.* [77] evaluated the antioxidant potential and the total phenolic content of the extracts of several species of brown seaweeds (*Ecklonia cava* Kjellman, *Ishige okamurae* Yendo, *Sargassum horneri* (Turner) C. Agardh, *Sargassum coreanum* J. Agardh, *Sargassum thunbergii* (Mertens ex Roth) Kuntze and *Scytosiphon lomentaria* (Lyngbye) J. Agardh) obtained by EAE with different enzymes, namely viscozyme, celluclast, AMG, termamyl, ultraflo, protamex, kojizyme, neutrase, flavourzyme, and alcalase. The extracts prepared by enzymatic hydrolysis of *E. cava* indicated strong free radical scavenging effects against DPPH (≈70% inhibition), except for ultraflo (29% inhibition), flavourzyme (32.6% inhibition) and alcalase (2.6% inhibition) extracts. However, some enzymatic extracts of *E. cava* and *S. coreanum* (especially alcalase extract) did not possess DPPH scavenging activity, although they contained as many phenolic compounds as the other extracts of *E. cava*, indicating that other compounds may be responsible for the scavenging activity. *E. cava* termamyl extract was the most effective one (68% of inhibition) against superoxide anion radicals, followed by all the enzymatic extracts from *S. horneri* (37.1%–58.6% inhibition), all of them more active than α-tocoferol, BHT and BHA. *S. fulvellum*, *S. thunbergii*, and *S. lomentaria* enzymatic extracts were also moderately active. The strongest potential was obtained against hydrogen peroxide. The highest activity was observed for *I. okamurae* kojizyme (≈96%), protamex and flavourzyme (93%) extracts, flavourzyme and alcalase extracts from *Sargassum thunbergii* (93%) and ultraflo and alcalase extracts of *S. horneri* (≈90%). Moreover, all the enzymatic extracts of *E. cava* displayed scavenging activity in the range of 60%–90%. Ultraflo and alcalase extracts of *S. horneri* were also able to inhibit DNA damage by the comet assay (approximately 50%). The potential of *E. cava* enzymatic extracts was analyzed in depth by Kim *et al.* [78] against hydrogen peroxide. Extracts were obtained with five carbohydrases (viscozyme, celluclast, AMG, termamyl, and ultraflo) and five proteases (protamex, kojizyme, neutrase, flavourzyme, and alcalase). The celluclast (≈2 g polyphenols/mL) and viscozyme extracts showed good hydrogen peroxide scavenging activities (73.25% and 72.92%, respectively), as compared to those obtained for other enzymatic extracts. The >30 kDa fraction of the celluclast extract also strongly enhanced cell viability against H_2_O_2_-induced oxidative damage and showed good lipid peroxidation inhibitory activity in a Chinese hamster lung fibroblast (V79-4) cell line. Other potentialities were encountered for the enzymatic extracts of *E. cava*. The kojizyme extract prepared with *E. cava* enhanced the proliferation of mice splenocytes and increased the number of their lymphocytes, monocytes, and granulocytes [181,182]; the polysaccharide fraction (richest in fucose and containing also rhamnose, galactose, glucose, mannose, and xylose) obtained from the AMG-assisted extraction from *E. cava* displayed the strongest anti-inflammatory activity in LPS-stimulated RAW 264.7 macrophages among several carbohydrases and proteases extracts [183]. More recently, the apoptotic effect of the neutrase extract of *S. coreanum*, as well as its fractions, was evaluated over several cancer cell lines (HL-60, CT-26, B-16, and HeLa cells) and normal cells (Vero cells), enlightening the promising properties of the crude polysaccharide fraction composed by fucose (40.85%), galactose (26.24%). and glucose (12.99%) against HL-60 cells [75].

The antioxidant potential of enzymatic extracts prepared from the red algae *Palmaria palmata* (L.) Weber and Mohr was tested using DPPH radical scavenging activity, oxygen radical absorbance capacity (ORAC), and ferrous ion-chelating ability assays [79]. All the proteases (umamizyme, alcalase, protamex, kojizyme, neutrase, and flavourzyme) tested, produced extracts with stronger effect on the extraction of polyphenols in comparison with the carbohydrases (viscozyme, ultraflo, AMG, celluclast, and termamyl) and cold water extraction. Umamizyme provided the highest extraction yield (76.3%), the highest total phenolic content (15.5 g gallic acid/kg extract) and the strongest scavenging capacity against DPPH (EC_50_ = 0.6 mg/mL) and peroxyl radical (>140 μmol trolox equivalents/g extract). The authors attributed the lower total phenolic content of carbohydrase extracts to the possible formation of protein–polyphenol complexes during extraction, which would prevent polyphenols release [79,184]. Afterwards, umamizyme extract was fractionated, revealing that while the crude polyphenol fraction possessed the highest peroxyl radical and DPPH scavenging activity, as demonstrated for the crude extract, the crude polysaccharide fraction was more effective as Fe^2+^ chelator [79].

Grimi *et al.* [185] studied the extraction of intracellular components from the microalgae *Nannochloropsis* sp. with application of two electrical and two mechanical cell disruption techniques, including PEF (20 kV/cm, 1–4 ms, 13.3–53.1 kJ/kg), high voltage electrical discharge (HVED) (40 kV/cm, 1–4 ms, 13.3–53.1 kJ/kg), UAE (200 W, 1–8 min, 12–96 kJ/kg), and high pressure homogenization (HPH) (1500 bar, 1–10 passes, 150–1500 kJ/kg). They concluded that PEF and HVED allowed selective extraction of water soluble ionic components and microelements, small molecular weight organic compounds (amino acids), and water soluble proteins, while UAE and HPH were more selective for pigments (chlorophylls or carotenoids) extraction.

Taurine is a sulfur-containing β-amino acid that cannot be easily synthesized in humans. It has several beneficial effects in the organisms, working as neurotransmitter, antioxidant, modulator of intracellular Ca^2+^, and as osmolyte [186]. Wang *et al.* [186] tested different UAE conditions to extract taurine from the red algae *Porphyra yezoensis* Ueda, namely extraction time (15–45 min), ultrasound power (100–300 W) and extraction temperature (20–60 °C). The solvent used was water. The highest taurine content was obtained at 300 W, 40.5 °C and 38.3 min.

Rudd and Benkendorff [187] extracted the brominated indole precursors of the dye Tyrian purple from the hypobranchial gland of the mollusc *Dicathais orbita* Gmelin using supercritical fluids (dried samples) and conventional chloroform:methanol extraction (dried and fresh samples). Tyrindoxyl sulfate was only present in the conventional extract obtained from the fresh material, while tyrindoleninone, 6-bromoisatin and tyriverdin were observed in the SFE extract (300 bar) and freeze-dried chloroform:methanol extract. At 150 bar, only 6-bromoisatin was detected, while at 500 bar the extact was dominated by tyrindoleninone and tyriverdin. These three compounds have important biological activities, such as anticancer and bacteriolytic. The great advantage of SFE extracts over conventional ones is the absence of choline esters, which can be toxic at high concentrations.

Byproducts (aquapharyngeal bulb and internal organs) of the sea cucumber *Cucumaria frondosa* Gunnerus were subjected to two different extraction methods. The traditional method consisted in successively extracting the dried material with hexane, acetone, MeOH, and water, while in the alternative one, the fresh material was incubated with one of the following enzymes: alcalase, neutral protease, papain, and protamex. All extracts were characterized in terms of crude protein, ash, fats, and neutral sugars, the extract obtained by the traditional extraction being the only one without neutral sugars. Papain extract obtained from aquapharyngeal bulb was the most active one against Herpes simplex virus type 1 (HSV-1). This extract was further fractionated and tested in Vero cells. The highest molecular weight fraction (>100 kDa) displayed the highest anti-HSV-1 activity, revealing a possible contribution of lectins to the inhibition of the virus [188]. The antiviral activity of enzymatic hydrolysates obtained from the seaweeds *Solieria chordalis* (C. Agardh) J. Agardh, *Ulva* sp. and *Sargassum muticulum* (Yendo) Fensholt was also studied by Hardouin *et al.* [189]. Six enzymes were used to hydrolyze the biomass, namely, two proteases and four carbohydrases, and the fractions obtained were characterized in terms of ash, neutral sugars, uronic acids, sulfate groups, proteins, total nitrogen lipids, and total phenol. The best anti-HSV-1 fraction tested on Vero cells was the one resulting from the action of endo-peptidase on *Solieria chordialis* dried biomass.

Fish bones are rich in chondroitin sulfate, which has a wide application in medicine, health food, cosmetics, and other fields due to its bioactivities, such as anticoagulant, antitumor, prevention of arteries hardening, and arthritis relieving [190]. He *et al.* [190] compared the extraction of chondroitin sulfate by PEF, UAE (23 min), EAE with pancreatic enzyme (240 min) and alkali method (120 min). PEF parameters evaluated were NaOH concentration (1%–6%), biomass:liquid ratio (1:5–1:25 g/mL), electric field strength (5–25 kV/cm) and pulse number (2–12). The optimized conditions were found to be electric field strength of 16.88 kV/cm, pulse number of nine, NaOH concentration of 3.24%, and biomass:liquid ratio of 1:15 g/mL. These conditions allowed 2.02, 1.84 and 1.42 times higher amount of chondroitin sulfateto be obtained than the EAE, alkali method and UAE, respectively, in a shorter time (5 min, pulse number =9, 18 μs).

**Table 10:** Advantages and disadvantages of the alternative methods [36,46,135,191].

	Advantages	Disadvantages
**MAE**	- Short treatment time and solvent consumption; - More efficient than conventional heating; - Reduction of extraction temperature using pressurized closed vessels; - Organic solvents and water can be used; - High extraction yields.	- Only solvents with high dielectric properties can be used; - Possible thermal degradation of the most thermolabile compounds when using open vessels; - High energy consumption.
**UAE**	- Short treatment time and solvent consumption; - High efficiency in cell disruption; - High extraction yields; - Suitable to extract thermolabile compounds; - Inexpensive.	- Solvents with low surface tension, low viscosity and low vapor pressure are preferable; - The presence of a dispersed phase contributes to theultrasound wave attenuation; - Ultrasounds generate heat, being important to accurately control the extraction temperature; - Excess of sonication may damage the quality of extracts.
**SFE**	- Green technology; - Higher selectivity because the solubility of a compound in a supercritical fluid can be manipulated; - Elimination of CO_2_ is achieved without residues, yielding a solvent-free extract; - Suitable to extract thermolabile compounds.	- High costs for the high pressure equipment needed; - The extraction of polar compounds requires the use of toxic modifiers (methanol, *etc.*); - Can be more time-consuming than the other alternative techniques.
**PSE**	- Green technology in the case of pressurized water extraction; - Reduced toxic solvent consumption; - Suitable to extract thermolabile compounds.	- High costs for the high pressure equipment needed; - Extractions performed at high temperatures may lead to degradation of thermolabile compounds.
**PEF**	- Green technology; - Short treatment time and increased yield due to cell membrane disruption; - The temperature increase is almost negligible; - The low-heat PEF treatment can minimize the degradation of heat-sensitive ingredients.	- Application of PEF processing is restricted to materials with no air bubbles and with low electrical conductivity.
**EAE**	- Water can be used (Green technology); - The enzyme treatment can increase the recovery of bioactive compounds.	- The efficiency of enzymatic hydrolysis is very low if plant materials have low moisture content; - Enzyme treatment is usually a slow process, and it may take from hours to days.
**SPS and SHS**	- They are easily removed from the bioactive compounds by bubbling CO_2_; - They are versatile.	- Very young technology, tests to assess their safety are needed.
**IL**	- They are very versatile, since their chemical and physical properties can be selected by choosing the cationic or the anionic constituents; - Safer technology because they are low-melting point, non-flammable and non-volatile solvents; - Some of them are environmentally friendly.	- Not all ILs are green solvents; - Some ILs require laborious purification.

## 4. Conventional *vs.* Alternative Extraction Methods: Future Perspectives

The selection of an appropriate extraction technique considers not only the degree of recovery, but also the cost, extraction time, volume of solvent used, selectivity, greenness of the method, and possibility of scale-up. All extraction methods, either the conventional or the alternative ones, present advantages and limitations (Table 10) [36,46,135,192].

According to Romanik *et al.* [34], Soxhlet was cheaper, but more time and solvent consuming than UAE, MAE, PSE and SFE. On the other hand, the implementation of high pressure equipment greatly increased the costs associated with PSE and SFE. Concerning to the time of extraction, it ranged from 6–48 h for Soxhlet extraction, less than 30 min by UAE, PSE and MAE and less than 60–120 min with SFE [34]. SFE, PSE, and extractions employing switchable solvents and ILs are very versatile and selective because they allow obtaining different extracts, either by using distinct conditions of pressure and temperature (SFE and PLE), different combinations of anion and cation (ILs) or by bubbling N_2_ or CO_2_ (Switchable solvents) [59,82,86].

In this new millennium, the search for environmentally friendly technologies has been a primary concern in laboratories devoted to the extraction of natural compounds. However, in what concerns marine organisms, the old fashion, toxic and exhaustive techniques are still the common practice around the world.

To change this paradigm it is necessary to prove the safety and efficacy of the new solvents, as well as to reduce the costs of the new pressurized technologies, or to increase the budget for research, in order to persuade researchers to invest in these new procedures. It is difficult to change when old procedures still work, but if the implementation of policies to reduce the use of toxic solvents is carried out, the alternative extraction methods will be more sought. In this sense, the European scenario is being dramatically altered by Regulation (EC) No 1907/2006—REACH (Registration, Evaluation, Authorisation and Restriction of Chemicals) [193], which concerns chemical substances, either as such, or incorporated into products or manufactured objects, whereas Directive 2008/1/EC—IPPC (Integrated Pollution Prevention Control) [194] is aimed at reducing the contribution of industry to non-sustainable development [40]. On the other hand, it is understandable that exhaustive extraction procedures, like Soxhlet extraction and maceration, are still used to extract marine compounds. In general, it is more challenging to obtain large quantities of bioactive compounds from marine organisms than from terrestrial species, since they produce only trace quantities of carotenoids, volatiles, sterols, alkaloids, *etc.* [195]. Moreover, the marine ecosystem is not inexhaustible and there is a supply problem that limits the use of large amount of marine organisms, especially of marine invertebrates [196]. Therefore, it is necessary to exhaustively extract with large amount of toxic solvents, to recover as much quantity of compounds and as many compounds as possible. New procedures are already available (as this review showed) that, despite being not so efficient, most of the time, are less time and solvent consuming and can avoid possible degradation, oxidation, or interconversion of the original compounds into different ones. For instance, SFE and PSE with apolar solvents are good options to extract volatile compounds (see Section 3.1.1), since it can be done at low temperatures. SFE is also a method of choice to selectively extract carotenoids to the detriment of chlorophylls (see Section 3.1.3), as well as to obtain a high unsaturated/saturated fatty acids ratio (Section 3.2), by simply choosing the adequate set of pressure and temperature. Regarding fatty acids, EAE with proteases can also contribute to enhance the extraction efficiency of fatty acids, since these enzymes hydrolyze the structural proteins in which fat globules are embedded [163]. In general, extraction of compounds from algae can take advantage of EAE with both proteases and carbohydrases, since they can degrade cell walls and release compounds [64], as well as from MAE, UAE and PEF, as these techniques work for the same purpose [32]. In what concerns other classes of compounds, few studies have been conducted and, thus, no conclusions about the best alternative extraction method can be withdraw.

We think that the new route will definitely be the choice for green technologies and for a sustainable use of marine ecosystems. However, the new millennium also brought the insistent claim of the “green label” to be attributed to new solvents and technologies. In 1998, Anastas and Warner [197] established the twelve principles of green chemistry, which concern: (1) the prevention of waste production; (2) atom economy; (3) less hazardous chemical syntheses; (4) the design of safer and less toxic chemicals; (5) the use of safer solvents and reduction of the use of auxiliary substances; (6) the design for energy efficiency; (7) the use of renewable feedstocks; (8) avoidance of unnecessary derivatization; (9) preference for catalytic reagents rather than stoichiometric reagents; (10) the design of degradation; (11) real-time analysis for pollution prevention; and (12) inherently safer chemistry for accident prevention.

Later, Chemat *et al.* [40] defined green extraction and established its six principles. Green extraction is based on the discovery and design of extraction processes that will reduce energy consumption, allow the use of alternative solvents and renewable natural products, and ensure a safe and high quality extract/product. Their principles are: (1) innovation by choosing renewable natural matrices; (2) the use of alternative and eco-friendly solvents; (3) reduction of energy consumption; (4) production of co-products instead of waste; (5) reduction of unit operations and favoring safer and controlled processes; (6) aiming for a non-denatured and biodegradable extract free from contaminants.

As a result of the competitiveness and the pressure of consumers for green products, there has been several inaccurate assertions of “greenness”, based only on one or a few principles of green chemistry [198]. For instance, ILs, in general, are claimed to be green solvents, but Deetlefs and Seddon [88] argued that some of them are not. Therefore, continued research on the safety/toxic assessment of new ILs is needed, as well as the development of new and green ILs. Nonetheless, SFE with CO_2_, PLE and other kinds of extractions performed with water correspond to a true green technology without the need of further evidence.

Another important factor to take into consideration is the possibility to scale-up the process from analytical to industrial scale, in order to extract large amounts of the compounds of interest and to convince industry to move to the alternative extraction techniques. Laboratory scale studies are used to obtain data on phase equilibria, mass transfer rate and solubility, which are necessary for the scaling up of the process [199]. Several works on the scale-up of SFE and hot water PSE have been published [191,200,201,202,203]. Conversely, for MAE and UAE more research is needed due to the scarce number of reports published [204,205]. However, large-scale extraction processes can only be performed when the supply problem is overcome, for example, by large-scale aquaculture.

## 5. Conclusions

This review compared different alternative methods for extracting marine bioactive compounds. Studies from the period comprising 2000–2014 reporting the extraction of terpenoids, fatty acids, sterols, alkaloids, phenolics, and other compounds were taken into consideration. From the analyzed data it can be concluded that, in comparison with traditional methods, alternative methods are much less time consuming, reducing the extraction time from hours to minutes. On the other hand, most of the time they are less efficient, allowing the recovery of lower amounts of bioactive compounds compared to the exhaustive Soxhlet extraction or maceration. However, if one’s goal is to reduce time and solvent consumption, alternative extraction methodologies are the best option.

Moreover, SFE and PLE are very sensitive methods, allowing the selective extraction of a determined class of compounds, to the detriment of others, by changing the set of pressure and temperature of the solvent (in SFE and PLE), or by adding a co-solvent (in the case of SFE) or by changing the solvent (in the case of PLE). The great advantage of these techniques is that in the case of CO_2_-SFE, the extracts are solvent-free and devoid of the toxic effects of hazard solvents. The same is applicable to PLE performed with dimethyl ether, which is a gas at normal pressure and temperature conditions.

The field of extraction of bioactive compounds has progressed with the advance of smart solvents. These amazing, powerful and highly selective solvents can be tailored to the matrix under study and a huge amount of combinations of ILs or SPS are nowadays available to selectively extract a particular class of compounds.

Finally, extraction yields can be greatly improved and the time of extraction greatly reduced by applying different techniques to disrupt cell walls and membranes. Mechanical (MAE and UAE), electrical (PEP) and enzymatic (EAE) technologies are available and results have proved their efficiency.

Despite all the advantages of the alternative extraction techniques described in this review, conventional extraction methods are still dominating the daily practices of most laboratories worldwide. This may be mainly because (i) of the costs associated with the implementation of high pressure techniques, such as SFE and PLE; (ii) the use of smart solvents is very recent and more tests to assess their safety are needed; (iii) the compounds extracted by alternative extraction can also be extracted with conventional methods.
